# CDK4/6-mediated phosphorylation of DUB3 promotes YAP1 stability and hepatocellular carcinoma progression

**DOI:** 10.1038/s41420-025-02493-x

**Published:** 2025-04-30

**Authors:** Lei Huang, Wenying Yuan, Xinying Li, Yixia Liu, Rui Wan, Xiuqing Ma, Tongzheng Liu, Junjie Liang, Yingjie Zhu

**Affiliations:** 1 College of Pharmacy / International Cooperative Laboratory of Traditional Chinese Medicine Modernization and Innovative Drug Development of Ministry of Education (MOE) of China, Jinan University, Guangdong, 510632 China; 2 Heze Traditional Chinese Medicine Hospital, Shandong, 274000 China; 3 Department of Hepatobiliary Surgery, The First Affiliated Hospital of Jinan University, Guangdong, 510630 China

**Keywords:** Hepatocellular carcinoma, Ubiquitylation, Targeted therapies, HIPPO signalling

## Abstract

Hepatocellular carcinoma (HCC) is one of the most lethal malignancies, frequently characterized by high expression and activation of Yes-associated protein 1 (YAP1), a key effector in the Hippo signaling pathway. Despite its crucial role in HCC progression, effective therapies directly targeting YAP1 remain challenging, underscoring the need to explore the regulatory mechanisms underlying its aberrant expression and activation. In this study, we identify cyclin-dependent kinase 4 and 6 (CDK4/6) as uncharacterized regulators of YAP1 in HCC. Genetic ablation or pharmacological inhibition of CDK4/6 significantly destabilizes YAP1 and attenuates its oncogenic functions both in vitro and in vivo. Furthermore, we establish DUB3 as a bona fide deubiquitinase of YAP1. Mechanistically, CDK4/6 directly phosphorylates DUB3, enhancing its deubiquitinase activity towards YAP1, which promotes tumor growth and contributes to chemo-resistance in HCC. Collectively, our findings unveil the previously unrecognized function and significance of the CDK4/6-DUB3 axis in stabilizing YAP1 and provide a rationale for potential therapeutic interventions in the treatment of HCC.

## Introduction

Hepatocellular carcinoma (HCC) accounts for more than 80% of primary liver cancers and among the most prevalent cancers worldwide, representing the third leading cause of cancer-related deaths [1]. Despite the significant advancements in treatment over the past decade, patient survival rates remain unsatisfactory due to factors such as unresectable disease at diagnosis, chemotherapy resistance, frequent recurrence, and limited treatment options. Although recent developments in molecular targeted therapy and immunotherapy have offered hope for some patients with advanced HCC, only a small subset of patients benefit from these treatments [2]. Thus, investigating the underlying molecular mechanism of HCC and developing novel therapeutic strategies are urgently needed.

The Hippo signaling pathway is an evolutionarily conserved pathway that plays crucial roles in organ development, tissue regeneration, immune regulation, wound healing, and epithelial homeostasis [3]. Under physiological conditions, Yes-associated protein 1 (YAP1) acts as a key downstream effector of the Hippo pathway. Phosphorylation by upstream core kinase cascades inactivates YAP1, leading to cytoplasmic retention and subsequent degradation. Conversely, when YAP1 is dephosphorylated, it translocates to the nucleus, where it interacts with the transcription factor TEADs to drive target genes (e.g. CYR61 and CTGF) that promote cell proliferation, migration, invasion, angiogenesis, and tumorigenesis [4, 5]. Dysregulation of Hippo signaling pathway is implicated in tumor growth, metastasis, and therapy resistance [6].

Numerous studies have linked elevated expression and activity of YAP1 to poor patient outcomes in various cancers, including HCC [7–9]. Furthermore, several in vitro and in vivo evidence supports the critical role of YAP1 in the development and progression of HCC [7, 10]. However, directly targeting YAP1 remains technically challenging [11], highlighting the necessity to explore upstream mechanisms regulating YAP1 stability and activation to identify potential druggable targets. Numerous studies, including ours, have shown that ubiquitination plays a significant role in the post-translational modification of YAP1 [12]. For instance, the E3 ubiquitin ligases Fbxw7 and β-TrCP have been implicated in YAP1 ubiquitination and degradation in HCC [13, 14], while the deubiquitinase USP19 enhances YAP1 stability [15]. Nevertheless, targeting these E3 ubiquitin ligases and deubiquitinase is challenging. And more druggable upstream regulators of YAP1 in cancers such as HCC are largely unknown and require further investigation.

In this study, we screened our in-house library of small molecule compounds and identified that Abemaciclib, a selective cyclin-dependent kinase 4 and 6 (CDK4/6) inhibitor approved by FDA for breast cancer treatment [16], as the strongest inhibitor of YAP1 activity. We demonstrated that CDK4/6 is crucial for maintaining YAP1 stability and its oncogenic functions in HCC. Additionally, we identify DUB3 as a previously unrecognized deubiquitinase of YAP1. Mechanistically, CDK4/6 directly binds to and phosphorylates DUB3, which leads to the deubiquitination and stabilization of YAP1, thereby promoting tumor growth and chemo-resistance of HCC. Taken together, our findings reveal the previously unrecognized tumor-promoting function of CDK4/6-DUB3 axis through stabilizing YAP1, providing preclinical evidence that targeting this axis may represent a promising therapeutic strategy for HCC.

## Results

### Identification of CDK4/6 as a critical regulator of YAP1 protein stability

**Fig. 1:**
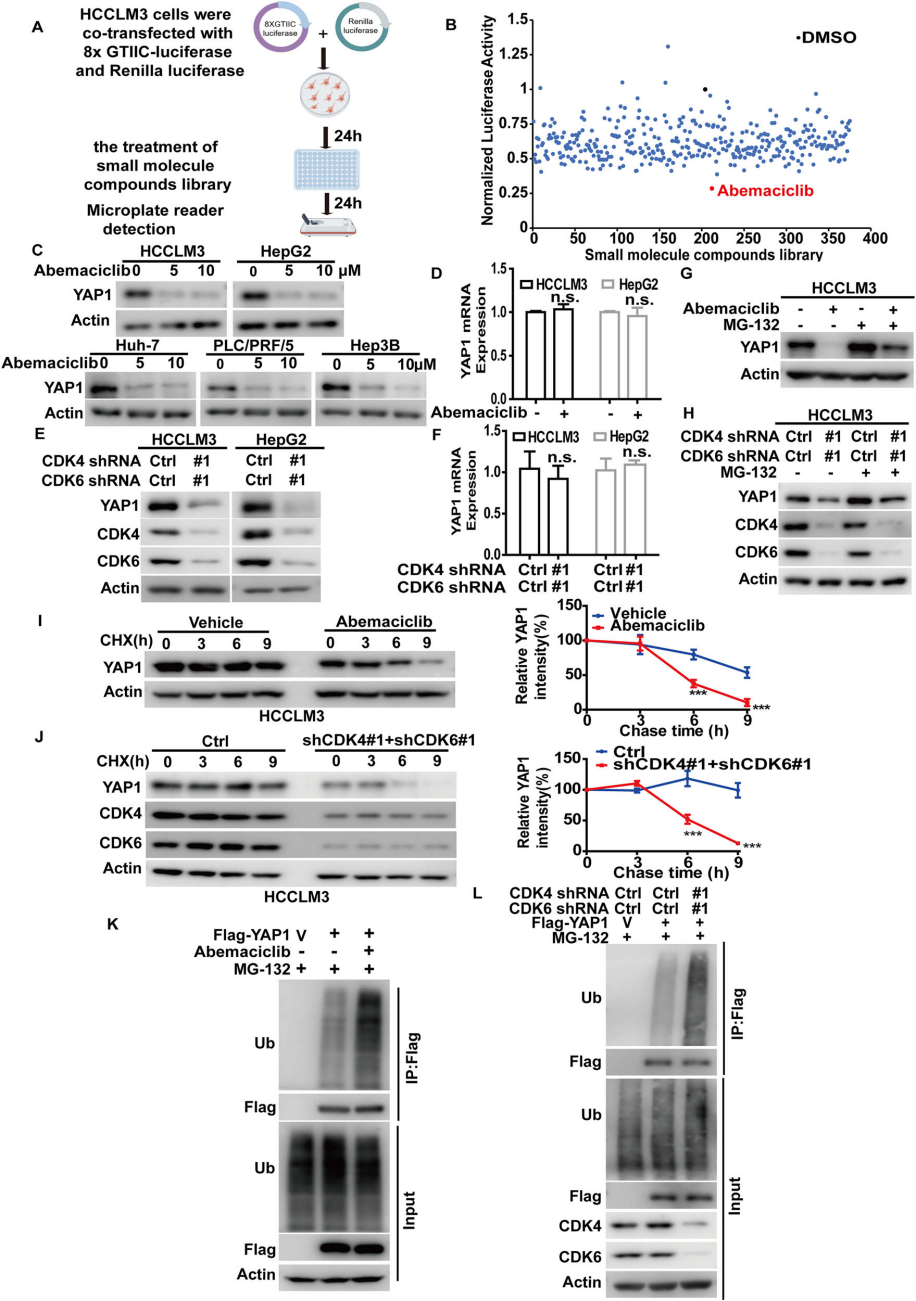
Identification of CDK4/6 as a critical regulator of YAP1 protein stability. **A** YAP1/TAZ-responsive (8×GTIIC) luciferase reporter assay was used to identify small molecule compounds that inhibit YAP1 activity. **B** Among the screened 374 in-house small molecule compounds, Abemaciclib, a highly selective CDK4/6 inhibitor, caused the highest inhibitory effect on YAP1 activity. The ratio of firefly to Renilla luciferase activity for each compound, as measured from triplicate assays, was normalized to the DMSO vehicle control. **C** HCCLM3, HepG2, Huh-7, PLC/PRF/5 and Hep3B cells were treated with Abemaciclib at indicated concentrations for 24 hours and western blot was performed with indicated antibodies. **D** Cells were treated with vehicle or Abemaciclib (5 μM) for 24 hours. Total RNA was isolated from the cells and *YAP1* mRNA level was determined by qRT-PCR. mRNA levels were determined relative to *GAPDH* and normalized relative to control. The results represent mean ± S.D. from three independent experiments; n.s., not significant. **E** HCCLM3 and HepG2 cells stably expressing control (Ctrl) or CDK4 shRNA#1 + CDK6 shRNA#1 were generated and western blot was performed with indicated antibodies. **F** Total RNA was isolated from cells in **E**. The expression of *YAP1* mRNA in cells was determined by qRT-PCR. Transcript levels were determined relative to *GAPDH* mRNA level and normalized relative to control. The results represent mean ± S.D. from three independent experiments; n.s., not significant. **G** HCCLM3 cells as in **D** were treated with either vehicle or MG-132 (10 μM) for an additional 24 hours. Western blot was performed with indicated antibodies. **H** HCCLM3 cells as in **E** were treated with vehicle or MG-132 and western blot was performed with indicated antibodies. **I** Cycloheximide pulse-chase assay was performed in cells as in **D**; the relative level of YAP1 to *β*-actin was measured by image J. The results represent mean ± S.D. from three independent experiments, ***p < 0.001. **J** Cycloheximide pulse-chase assay was performed in cells as in **E**; the relative level of YAP1 to *β*-actin was measured by image J. The results represent mean ± S.D. from three independent experiments; ***p < 0.001. **K** Cells were transfected with vector (V) or Flag-YAP1 and then treated with vehicle or Abemaciclib for 24 hours in the presence of MG-132 (10 μM). Cell lysates were immunoprecipitated with anti-Flag affinity gel, and the polyubiquitylated YAP1 was detected by anti-ubiquitin antibody. **L** Cells stably expressing control or CDK4/6 shRNAs were transfected with indicated plasmids and treated with MG-132 (10 μM) for 10 hours. Cell lysates were subjected to immunoprecipitation with anti-Flag affinity gel and the ubiquitination of YAP1 protein was examined by western blot.

YAP1, a potent transcriptional coactivator and a key downstream effector of Hippo pathway, is implicated in tumor pathogenesis and is frequently activated in various cancers including HCC [17, 18]. To confirm the oncogenic role of YAP1 in HCC, we demonstrated that YAP1 knockdown significantly reduced the proliferation of HCCLM3 and HepG2 cells, while YAP1 overexpression promoted cell proliferation (Fig. S1A-F). Given the challenges in directly targeting deregulated YAP1 in caners, we performed the YAP1/TAZ-responsive (8×GTIIC) luciferase reporter assay to screen our in-house library of small molecule compounds for potential inhibitors of YAP1 activity in HCCLM3 cell line (Fig. 1A). Among the screened 374 small molecule compounds, Abemaciclib, a highly selective CDK4/6 inhibitor approved by FDA for breast cancer treatment [16], exhibited the strongest inhibitory effect, reducing YAP1 activity by more than 70% (Fig. 1B).

This result prompts us to investigate the underlying mechanism of CDK4/6 to regulate YAP1. As shown in Fig. 1C–F, co-knockdown of CDK4/6 or pharmacological inhibition by Abemaciclib in HCC cells significantly reduced YAP1 protein levels without affecting its mRNA level. Intriguingly, treatment of proteasome inhibitor MG-132 could rescue the decrease in YAP1 protein levels in CDK4/6-depleted cells or cells treated with Abemaciclib, indicating a proteasome-dependent mechanism (Figs. 1G, H and S2A-B). Additionally, results from cycloheximide pulse-chase assay showed that YAP1 protein was less stable in cells depleting CDK4/6 or treated with Abemaciclib (Figs. 1I, J and S2C-D), which could be due to the increased ubiquitination level of YAP1 (Fig. 1K, L). We also found that the protein levels of TAZ, which shares high homology with YAP1 and similarly functions as a downstream effector of the Hippo pathway [4], were reduced in CDK4/6-deficient cells or cells treated with Abemaciclib due to increased TAZ polyubiquitination. Furthermore, co-knockdown of CDK4/6 or treatment with Abemaciclib markedly decreased the expression of CYR61 and CTGF, two target genes of YAP1 (Fig. S3A-D). Together, these findings suggest that CDK4/6 is a critical upstream regulator of YAP1 stability in HCC cells.

### CDK4/6 inhibition suppresses HCC progression through destabilizing YAP1

**Fig. 2:**
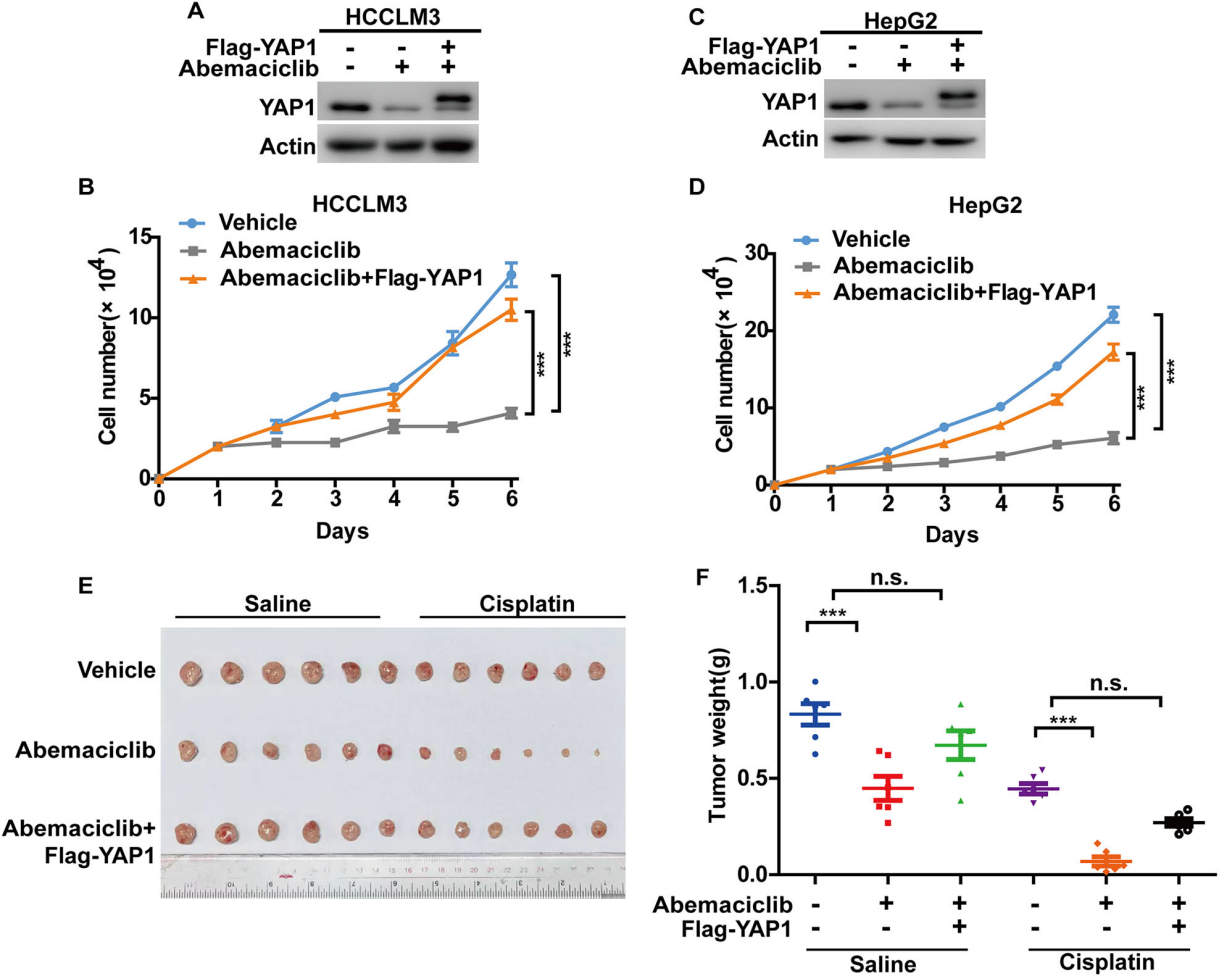
CDK4/6 inhibition suppresses hepatocellular carcinoma progression through YAP1. **A** HCCLM3 cells were transfected with indicated plasmids and treated with vehicle or Abemaciclib. Western blot was performed with indicated antibodies. **B** Cell proliferation assay was performed from cells in **A**. Results represent the mean ± S.D. of three independent experiments; ***p < 0.001, Vehicle vs Abemaciclib, Abemaciclib + Flag-YAP1 vs Abemaciclib. **C** HepG2 cells were transfected with indicated plasmids and treated with vehicle or Abemaciclib. Western blot was performed with indicated antibodies. **D** Cell proliferation assay was performed from cells in **C**. Results represent the mean ± S.D. of three independent experiments; ***p < 0.001, Vehicle vs Abemaciclib, Abemaciclib + Flag-YAP1 vs Abemaciclib. **E, F** Cells as in **A** were subcutaneously implanted into nude mice (*n* = 6 per group). When tumors reached around 150 mm^3^ in size, mice were administered with saline, Abemaciclib (75 mg/kg, oral gavage 4 days a week for 4 weeks), cisplatin (5 mg/kg intraperitoneal twice per week) or the combination of Abemaciclib and cisplatin. Tumors were collected (**E**) and weights were measured (**F**). Results represent the mean ± S.D. of data from six mice; n.s., not significant, ***p < 0.001.

We next investigated whether targeting CDK4/6 could destabilize YAP1 and inhibit its oncogenic effects in HCC. As shown in Fig. 2A–D, pharmacological inhibition of CDK4/6 by Abemaciclib significantly inhibited cell proliferation of HCCLM3 and HepG2 cells. Notably, reconstitution of YAP1 expression markedly rescued this inhibitory effect, confirming the role of YAP1 in mediating the response to CDK4/6 inhibition. In the line with these in vitro results, xenograft animal studies showed that Abemaciclib effectively inhibited tumor growth and increased the sensitivity to cisplatin. Importantly, this tumor growth inhibition was largely abrogated by the reconstitution of YAP1 expression (Fig. 2E, F). These results indicate that inhibition of CDK4/6 suppresses tumor progression of HCC, at least in part through destabilizing YAP1.

### Identification of DUB3 as a bona fide deubiquitinase of YAP1

**Fig. 3:**
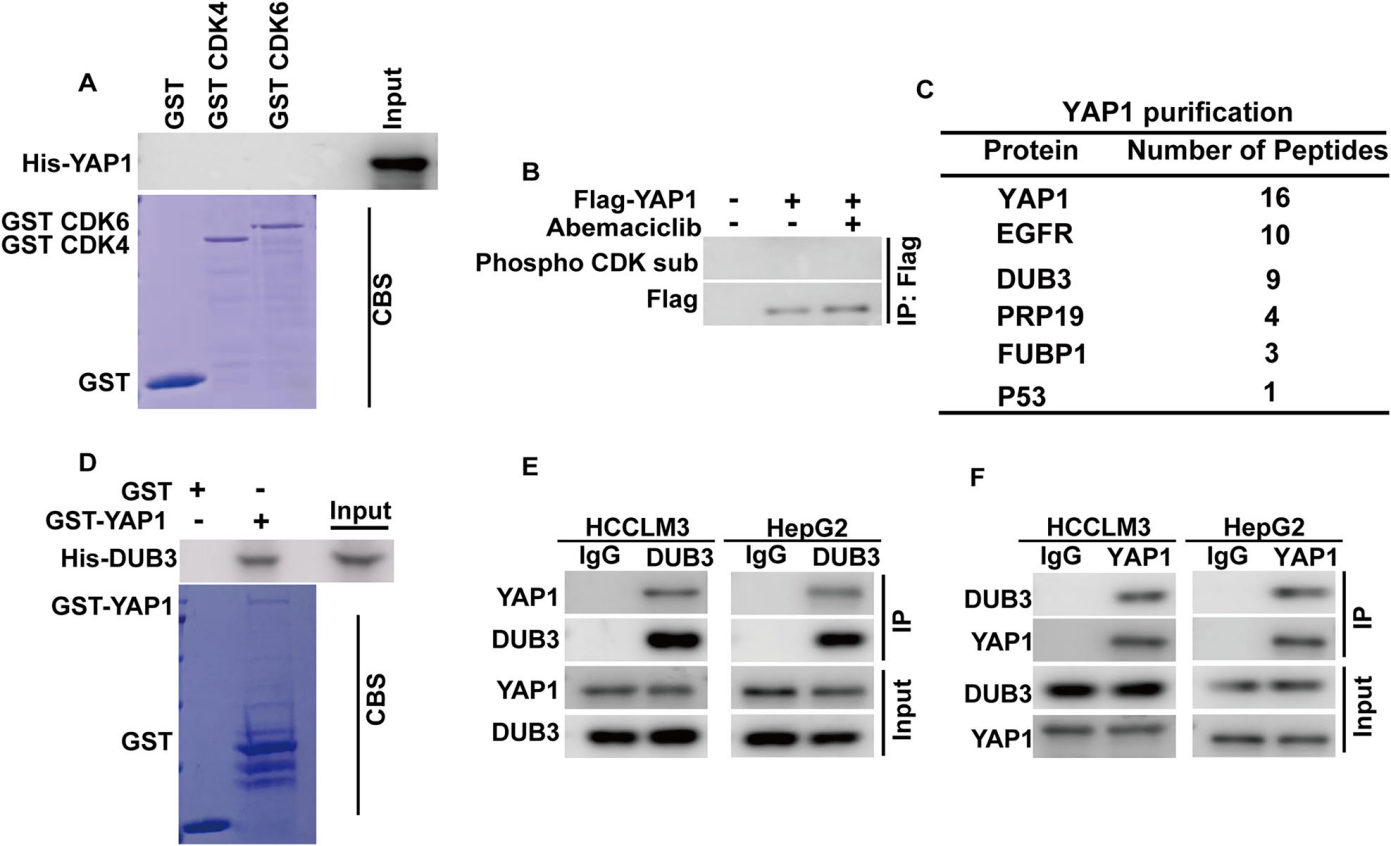
DUB3 interacts with YAP1. **A** Purified recombinant GST, GST-CDK4, GST-CDK6 and His-YAP1 were incubated in vitro and the interaction between CDK4/6 and YAP1 was examined. CBS, Coomassie blue staining. **B** HCCLM3 cells stably expressing vector or Flag-YAP1 were generated and treated with vehicle or Abemaciclib. Cell lysates were subjected to immunoprecipitation with anti-Flag affinity gel and the phosphorylation of YAP1 were examined by phospho-CDK substrate antibody. **C** List of YAP1-associated proteins identified by mass spectrometric analysis. HCCLM3 cells stably expressing Flag-YAP1 were generated and YAP1 complexes were subjected to mass spectrometric analysis. **D** Purified recombinant GST, GST-YAP1 and His-DUB3 were incubated in vitro and the interaction between YAP1 and DUB3 was examined. CBS, Coomassie blue staining. **E** HCCLM3 and HepG2 cell lysates were subjected to immunoprecipitation with IgG or anti-DUB3 antibody. The immunoprecipitates were blotted with indicated antibodies. **F** HCCLM3 and HepG2 cell lysates were subjected to immunoprecipitation with IgG or anti-YAP1 antibody. The immunoprecipitates were blotted with indicated antibodies.

CDK4/6 has been reported to phosphorylate and regulate the stability of various substrates such as FOXM1 [19]. Surprisingly, our attempts to demonstrate a direct interaction between bacterial-purified GST-CDK4, GST-CDK6, and His-YAP1 in vitro did not yield detectable results, nor could we observe the phosphorylation of YAP1 using a CDK-substrate specific antibody (Fig. 3A, B). These findings suggest that YAP1 might not be a direct substrate of CDK4/6 and that an unidentified intermediary factor may mediate the regulation of YAP1 by CDK4/6.

To identify the potential linker between CDK4/6 and YAP1, we performed tandem affinity purification and mass spectrometry analysis using HCCLM3 cells stably expressing Flag-YAP1. As shown in Fig. 3C, alongside several known YAP1-interacting proteins such as EGFR [20] and P53 [21], we identified the deubiquitinase DUB3 as a major interactor of YAP1. The direct interaction between purified GST-YAP1 and His-DUB3 was also detected (Fig. 3D), indicating that DUB3 could directly interact with YAP1. Furthermore, we validated the endogenous interaction between DUB3 and YAP1 in both HCCLM3 and HepG2 cells (Fig. 3E, F).

### DUB3 deubiquitinates and stabilizes YAP1

**Fig. 4:**
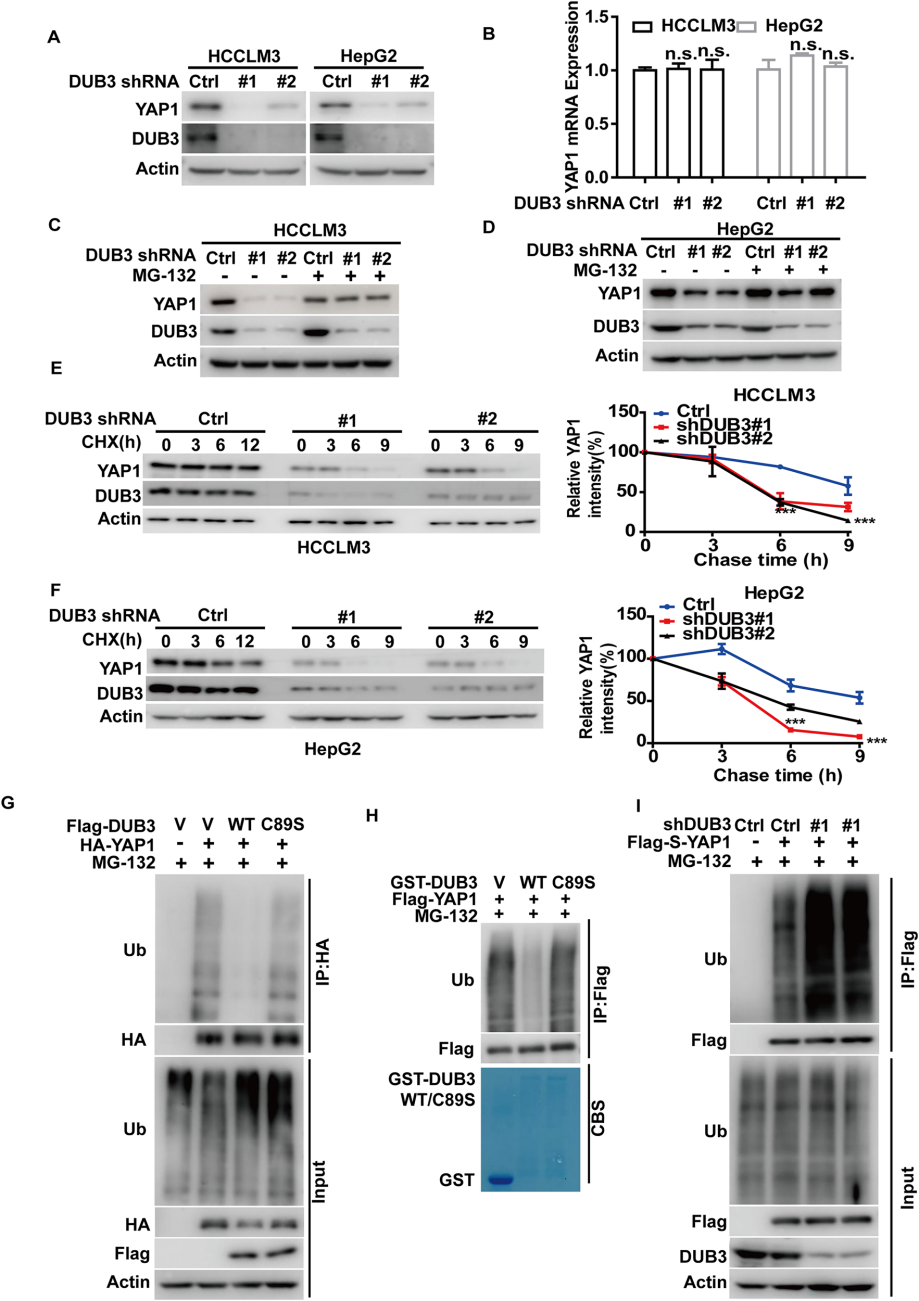
DUB3 deubiquitinates and stabilizes YAP1. **A** HCCLM3 and HepG2 cells stably expressing control (Ctrl) or DUB3 shRNAs (#1 and #2) were generated and western blot was performed with indicated antibodies. **B** Total RNA was isolated from cells in **A**. The expression of *YAP1* mRNA in cells was determined by qRT-PCR. Transcript levels were determined relative to *GAPDH* mRNA level and normalized relative to control. The results represent mean ± S.D. from three independent experiments; n.s., not significant. **C** HCCLM3 cells as in **A** were treated with vehicle or MG-132 and western blot was performed with indicated antibodies. **D** HepG2 cells as in **A** were treated with vehicle or MG-132 and western blot was performed with indicated antibodies. **E** Cycloheximide pulse-chase assay was performed in HCCLM3 cells as in **A**; the relative level of YAP1 to *β*-actin was measured by image J. The results represent mean ± S.D. from three independent experiments; ***p < 0.001. **F** Cycloheximide pulse-chase assay was performed in HepG2 cells as in **A**; the relative level of YAP1 to *β*-actin was measured by image J. The results represent mean ± S.D. from three independent experiments; ***p < 0.001. **G** 293 T Cells were transfected with vector (V), HA-YAP1, Flag-DUB3 WT or Flag-DUB3 C89S mutant as indicated, then were treated with MG-132 for 10 hours. Cell lysates were immunoprecipitated with HA-protein agaroses and the polyubiquitylated YAP1 protein was detected by anti-ubiquitin antibody. **H** Cells were transfected with Flag-YAP1 and treated with MG-132 (10 μM) for 10 hours. Cell lysates were immunoprecipitated with anti-Flag affinity gel and incubated with purified GST, GST-DUB3 WT or GST-DUB3 C89S mutant in a cell-free condition. The polyubiquitylated YAP1 protein was detected by anti-ubiquitin antibody. CBS, Coomassie blue staining. **I** 293 T cells stably expressing control or DUB3 shRNA were transfected with vector or Flag-S-YAP1 as indicated, then were treated with MG-132 for 10 hours. Cell lysates were immunoprecipitated with anti-Flag affinity gel and the polyubiquitylated YAP1 protein was detected by anti-ubiquitin antibody.

The direct interaction of DUB3 and YAP1 prompted us to examine the potential role of DUB3 in regulating YAP1 in HCC. We found that depletion of DUB3 in HCCLM3 and HepG2 cells significantly decreased YAP1 protein levels (Fig. 4A), but did not affect *YAP1* mRNA levels (Fig. 4B). Furthermore, treatment of MG-132 rescued the decreased YAP1 protein levels in DUB3-deficient cells (Fig. 4C, D). Consistent with this observation, YAP1 protein was less stable in DUB3-depleted cells than control assessed by cycloheximide pulse-chase assay (Fig. 4E, F). To further elucidate the role of DUB3, we performed ubiquitination assays under different conditions. Our results showed that DUB3 WT, but not its catalytically inactive mutant C89S significantly decreased the ubiquitination level of YAP1 (Fig. 4G). We next confirmed this finding in vitro and observed that purified GST-DUB3 WT significantly reduced polyubiquitinated YAP1 levels, whereas the C89S mutant had no such effect (Fig. 4H). Additionally, depletion of DUB3 markedly increased the ubiquitination level of YAP1 (Fig. 4I). The TAZ protein level in HCCLM3 cells with DUB3 depletion was also dramatically decreased, likely due to altered polyubiquitination. We further examined the downstream target genes of YAP1 and found that DUB3 depletion resulted in significant reductions in CYR61 and CTGF protein levels (Fig. S4A-B). Taken together, these results demonstrate that DUB3 functions as a bona fide deubiquitinase for YAP1 in HCC, facilitating its deubiquitination and stabilization.

### DUB3 promotes tumor progression of HCC through stabilizing YAP1

**Fig. 5:**
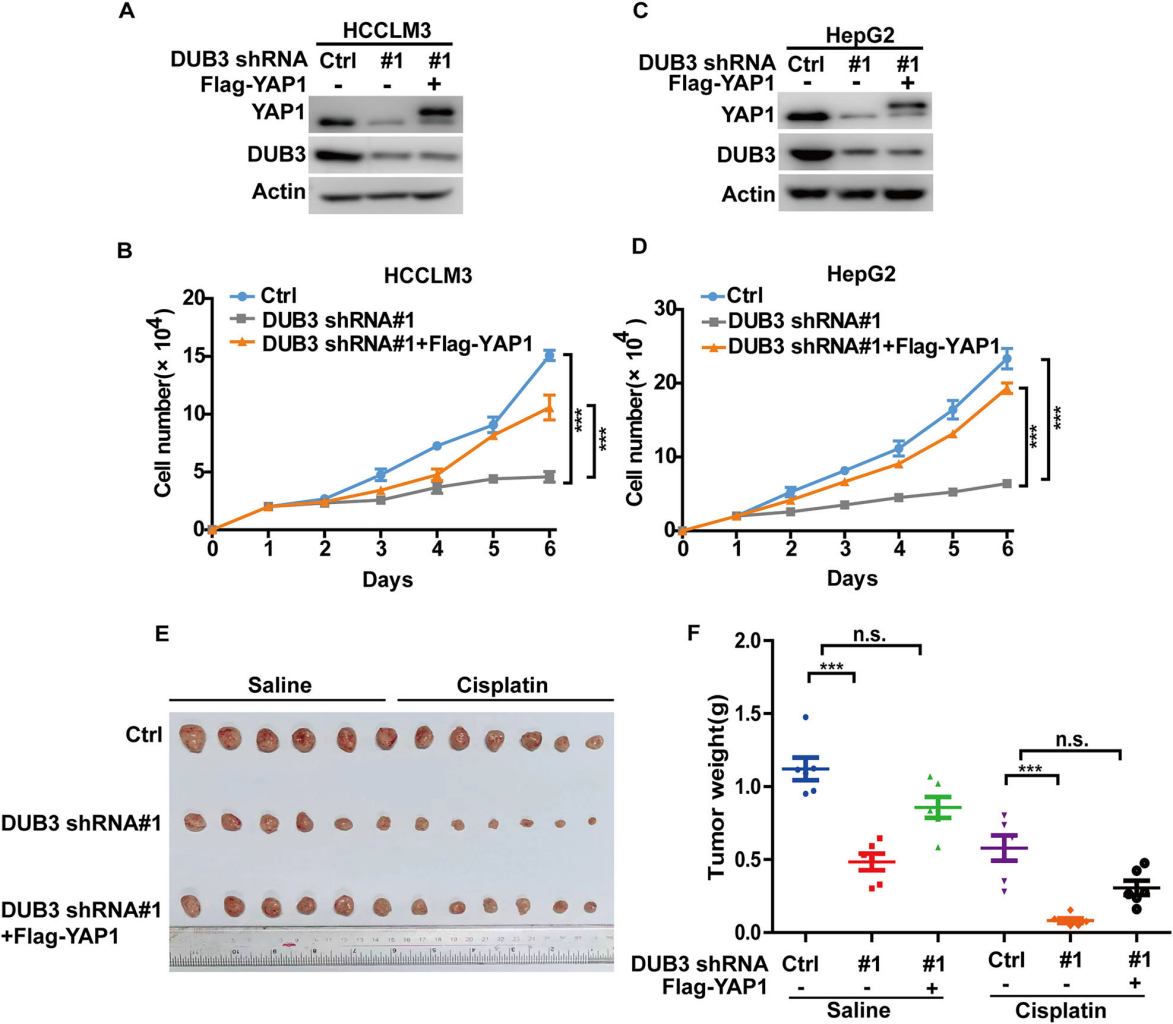
DUB3 promotes hepatocellular carcinoma progression through stabilizing YAP1. **A** HCCLM3 cells stably expressing control (Ctrl) or DUB3 shRNA#1 were transfected with vector or Flag-YAP1 and western blot was performed with indicated antibodies. **B** Cell proliferation assay was performed from cells in **A**. Results represent the mean ± S.D. of three independent experiments; ***p < 0.001, Ctrl vs DUB3 shRNA #1, DUB3 shRNA #1 + Flag-YAP1 vs DUB3 shRNA #1. **C** HepG2 cells stably expressing control or DUB3 shRNA#1 were transfected with vector or Flag-YAP1 and western blot was performed with indicated antibodies. **D** Cell proliferation assay was performed from cells in **C**. Results represent the mean ± S.D. of three independent experiments; ***p < 0.001, Ctrl vs DUB3 shRNA #1, DUB3 shRNA #1 + Flag-YAP1 vs DUB3 shRNA #1. **E, F** Cells as in **A** were subcutaneously implanted into nude mice (*n* = 6 per group) and mice were treated with saline or cisplatin (2 mg/kg). Tumors were collected (**E**) and weights were measured (**F**). The results represent the mean ± S.D. of data from six mice; n.s., not significant, ***p < 0.001.

We next investigated whether DUB3 could promote HCC progression in a YAP1-dependent manner. As shown in Fig. 5A–D, depletion of DUB3 in HCCLM3 and HepG2 cells significantly reduced cell proliferation. However, this effect could be largely restored by reconstitution of YAP1 in DUB3-depleted cells. Consistent with these in vitro findings, results from xenograft animal studies showed that knockdown of DUB3 inhibited tumor growth and increased sensitivity to cisplatin treatment. Notably, reconstitution of YAP1 markedly reversed such an inhibitory effect (Fig. 5E, F). Altogether, these results indicate that DUB3 is crucial for HCC progression through stabilizing YAP1.

### Phosphorylation of DUB3 by CDK4/6 promotes YAP1 stability and oncogenic function

**Fig. 6:**
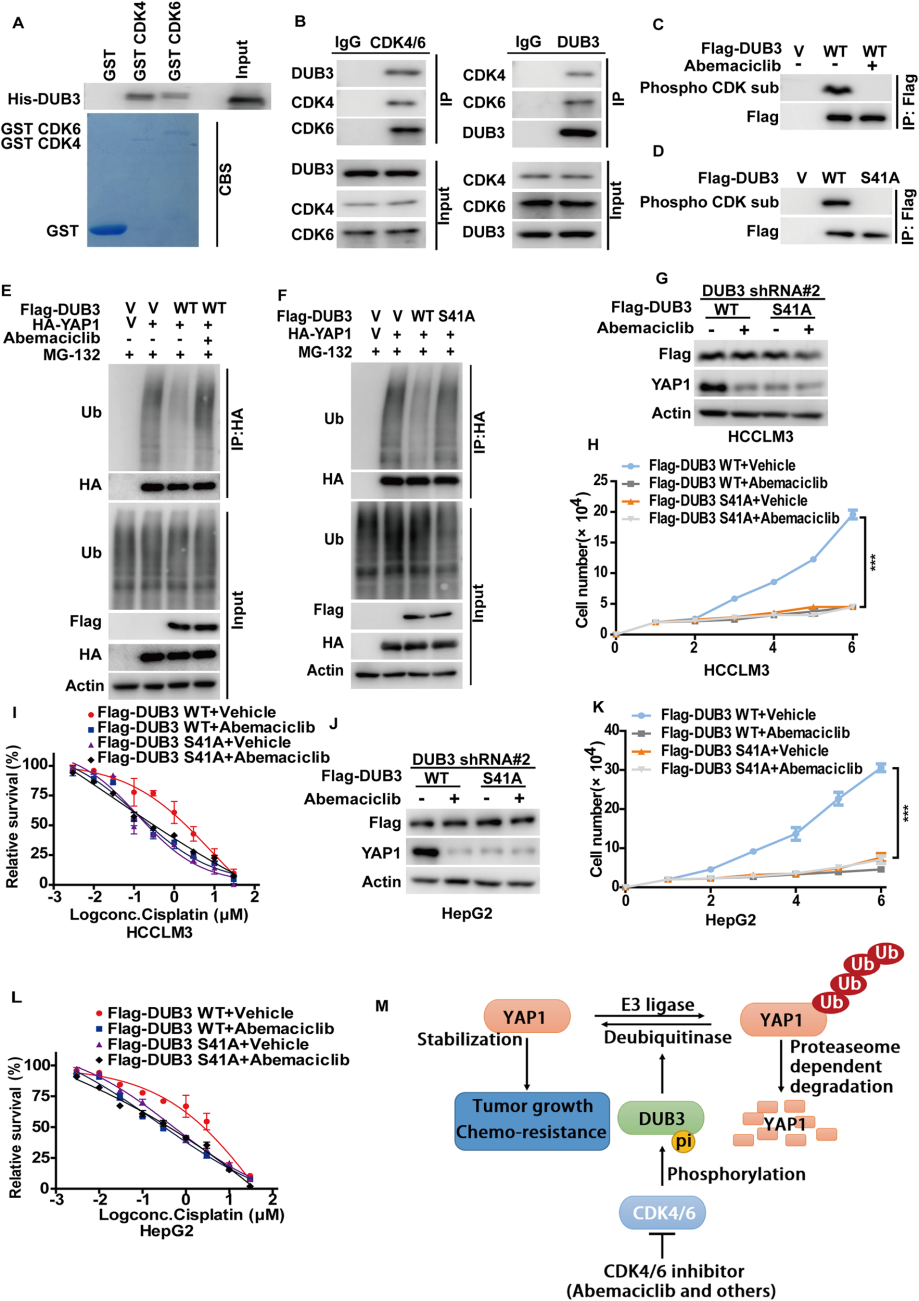
Phosphorylation of DUB3 by CDK4/6 regulates YAP1 stability and its oncogenic function in hepatocellular carcinoma. **A** Purified recombinant GST, GST-CDK4, GST-CDK6 and His-DUB3 were incubated in vitro and the interaction between CDK4/6 and DUB3 was examined. CBS, Coomassie blue staining. **B** HCCLM3 cell lysates were subjected to immunoprecipitation with IgG, anti-CDK4/6 or anti-DUB3 antibodies. The immunoprecipitates were blotted with indicated antibodies. **C** HCCLM3 cells were transfected with vector (V) or Flag-DUB3 WT as indicated and treated with vehicle or Abemaciclib. Cell lysates were subjected to immunoprecipitation with anti-Flag affinity gel and the phosphorylation of DUB3 were examined by phospho-CDK substrate antibody. **D** Empty vector (V) or Flag-DUB3 (WT or S41A mutant) were transfected in HCCLM3 cells. Cell lysates were subjected to immunoprecipitation with anti-Flag affinity gel and the phosphorylation of DUB3 were examined by phospho-CDK substrate antibody. **E** Cells were transfected with indicated plasmids and treated with vehicle or Abemaciclib. Cell lysates were immunoprecipitated with HA-protein agaroses and the polyubiquitylated YAP1 protein was detected by anti-ubiquitin antibody. **F** Cells were transfected with vector, HA-YAP1, Flag-DUB3 WT or Flag-DUB3 S41A mutant as indicated, then were treated MG-132 for 10 hours. Cell lysates were immunoprecipitated with HA-protein agaroses and the polyubiquitylated YAP1 protein was detected by anti-ubiquitin antibody. **G** HCCLM3 cells stably expressing DUB3 shRNA#2 were transfected with indicated plasmids and treated with vehicle or Abemaciclib, western blot was performed with indicated antibodies. **H** Cell proliferation assay was performed from cells in **G**. Results represent the mean ± S.D. of three independent experiments; ***p < 0.001, Flag-DUB3 WT + Vehicle vs Flag-DUB3 WT + Abemaciclib, Flag-DUB3 WT + Vehicle vs Flag-DUB3 S41A + Vehicle, Flag-DUB3 WT + Vehicle vs Flag-DUB3 S41A + Abemaciclib. **I** Cells as in **G** were treated with indicated concentrations of cisplatin and cell survival was determined. The results represent mean ± S.D. of three independent experiments. **J** HepG2 cells stably expressing DUB3 shRNA were transfected with indicated plasmids and treated with vehicle or Abemaciclib, western blot was performed with indicated antibodies. **K** Cell proliferation assay was performed from cells in **J**. Results represent the mean ± S.D. of three independent experiments; ***p < 0.001, Flag-DUB3 WT + Vehicle vs Flag-DUB3 WT + Abemaciclib, Flag-DUB3 WT + Vehicle vs Flag-DUB3 S41A + Vehicle, Flag-DUB3 WT + Vehicle vs Flag-DUB3 S41A + Abemaciclib. **L** Cells as in **J** were treated with indicated concentrations of cisplatin and cell survival was determined. The results represent mean ± S.D. of three independent experiments. **M** The working model to illustrate that CDK4/6-mediated phosphorylation of DUB3 regulates tumor growth and chemo-resistance through YAP1.

Given that both CDK4/6 and DUB3 contribute to tumor progression in HCC by stabilizing YAP1, we hypothesized that DUB3 might serve as a linker between CDK4/6 and YAP1. Our previous study established that CDK4/6 binds to and phosphorylates DUB3 at Ser41 [22]. As shown in Fig. 6A, we observed a clear direct interaction between purified GST-CDK4 or GST-CDK6 and His-DUB3. We validated the endogenous interaction between CDK4/6 and DUB3 in HCCLM3 cells (Fig. 6B). Furthermore, both CDK4/6 inhibitor Abemaciclib and the DUB3 S41A mutant significantly attenuated the phosphorylation of DUB3 WT, as detected by a CDK-substrate specific antibody (Fig. 6C, D).

We next investigated whether CDK4/6-mediated phosphorylation of DUB3 affects YAP1 stability and its oncogenic functions. DUB3 WT dramatically reduced the ubiquitination level of YAP1 and consequently stabilized YAP1, which were markedly mitigated by the pretreatment with Abemaciclib (Fig. 6E). In contrast, the DUB3 S41A mutant hardly influenced the YAP1 ubiquitination level (Fig. 6F). As shown in Fig. 6G–L, reconstitution of DUB3 WT, but not the DUB3 S41A mutant, in endogenous DUB3-deficient HCCLM3 and HepG2 cells dramatically increased YAP1 protein levels, enhanced cell proliferation as well as decreased sensitivity to cisplatin. Furthermore, treatment with Abemaciclib markedly mitigated YAP1 protein levels and exhibited a substantial tumor-suppressive effect in HCC cells reconstituted with DUB3 WT. These results indicate that CDK4/6-mediated phosphorylation of DUB3 is pivotal for YAP1 stability and its oncogenic function in HCC. Overall, our study demonstrates that the CDK4/6-DUB3 axis contributes to the stabilization of YAP1, presenting an attractive therapeutic strategy in the treatment of HCC (Fig. 6M).

## Discussion

HCC is the most prevalent form of primary liver cancers, characterized by high malignancy and mortality rate. Despite advances in diagnostic and therapeutic strategies, effective targeted therapies for HCC remain limited, resulting in unsatisfactory overall survival and prognosis for patients [23]. Therefore, elucidating unidentified molecular mechanisms and identifying potential intervention targets are crucial for developing innovative treatment strategies for HCC.

The elevated expression and activity of YAP1, a pivotal downstream effector of the Hippo pathway, are associated with tumor growth, metastasis, therapy resistance, and poor prognosis in various human cancers, including HCC [7, 8, 17, 24, 25]. Consistent with previous studies, our results confirm the oncogenic role of YAP1 in HCC (Fig. S1), suggesting that targeting YAP1 may offer a promising therapeutic avenue. However, the lack of significant binding pockets in YAP1 complicates the development of pharmacological agents aimed to directly targeting YAP1 [11]. Additionally, recent approaches exploring small molecule inhibitors to disrupt the YAP1-TEAD interaction, such as Verteporfin, have demonstrated promising preclinical antitumor activity [26, 27], but have shown limited clinical efficacy due to off-target toxicity in human cancers [28]. Therefore, identifying upstream regulatory mechanisms of YAP1 could provide alternative strategies for discovering more druggable targets for YAP1-based therapies. In this study, we unveil the previously uncharacterized tumor-promoting function and clinical significance of the CDK4/6-DUB3 axis in stabilizing YAP1, providing preclinical evidence that targeting CDK4/6 and DUB3 constitutes an effective strategy for suppressing tumor growth and overcoming chemo-resistance in HCC (Fig. 6M).

CDK4/6 play a vital role as key regulators of the G1-to-S phase transition in the cell cycle, contributing significantly to the initiation and progression of various malignancies [29, 30]. With the emergence of CDK4/6 inhibitors as anti-cancer drugs, three selective CDK4/6 inhibitors Palbociclib, Ribociclib and Abemaciclib are approved for breast cancer treatment [31, 32]. Increasing evidence suggests that CDK4/6 inhibitors hold significant promise in the treatment of other cancers such as non-small cell lung cancer [33], esophageal cancer [34], and pancreatic ductal adenocarcinoma [35]. For instance, CDK4/6 inhibitor suppresses cellular growth, induces apoptosis, and also inhibits migration, invasion and metastasis in esophageal squamous cell carcinoma [36]. A previous study has reported that CDK 4/6 inhibitor triggers an RB-dependent SMAC-mediated apoptotic response, leading to the inhibition of tumor growth in non-small cell lung cancer [37]. However, the downstream mechanisms by which CDK4/6 inhibitors exert their antitumor effects in HCC remain largely unexplored. Here, our study reveals the unrecognized function of CDK4/6 in stabilizing YAP1, as supported by the following evidence. We screened our in-house library of small molecule compounds and identified Abemaciclib as the most potent inhibitor of YAP1 activity (Fig. 1A, B). Notably, pharmacological inhibition of CDK4/6 using Abemaciclib or genetic depletion of CDK4 and CDK6 in HCC cells significantly decreases the protein levels of YAP1 and its downstream targets, such as CYR61 and CTGF, without affecting *YAP1* mRNA levels (Figs. 1C–F and S3). Next, we demonstrate that CDK4/6 promotes YAP1 protein stability, as evidenced by the increased ubiquitination level and shortened half-life of YAP1 in cells depleting CDK4/6 or treated with Abemaciclib (Figs. 1C–F and S2). Importantly, Abemaciclib not only markedly suppresses cell proliferation in vitro but also inhibits tumor growth and enhances chemosensitivity in vivo, which are largely reversed by reconstituting YAP1 expression (Fig. 2). Since the reconstitution of YAP1 could not completely restore the malignant phenotypes caused by the inhibition of CDK4/6 in HCC cells, it is noteworthy that we cannot rule out the possibility of additional mechanisms involved in CDK4/6-mediated tumor progression of HCC. In lung adenocarcinoma [38] and breast cancer [39], CDK4/6-mediated phosphorylation and activation of USP51 is essential for deubiquitinating and stabilizing ZEB1, thereby promoting cancer metastasis. CDK4/6-E2F1 mediates an increase in MAGED1 expression, which promotes FBP1 degradation and the Warburg effect in pancreatic cancer [40]. Hence, the potential involvements of other substrates of CDK4/6 besides YAP1 in tumor progression of HCC need to be further investigated.

Intriguingly, our results indicate that YAP1 is not the direct substrate of CDK4/6, as neither direct interaction of purified GST-CDK4, GST-CDK6 with His-YAP1 nor CDK-mediated phosphorylation of YAP1 was detected (Fig. 3A, B). Instead, our findings reveal that the deubiquitinase DUB3 functions as a novel intermediary linking CDK4/6 to YAP1. DUB3 is implicated in various biological processes, including DNA damage response [41], epithelial-mesenchymal transition [22], cell proliferation [42], and cell cycle progression [43]. Recently, DUB3 has been demonstrated to promote breast cancer metastasis and hold the therapeutic potential for breast cancer [22, 44]. Our previous study has also established that CDK4/6 phosphorylates DUB3 at Ser41, leading to the stabilization of Snail1 and promoting triple negative breast cancer metastasis [22]. However, the role of DUB3 in HCC remains controversial. Jia et al. have reported that DUB3 inhibits HCC cell proliferation in vitro and tumor growth in vivo while enhancing the chemosensitivity of HCC cells in a KLF4-dependent manner [45]. In contrast, a recent study has shown that NXN suppresses the proliferation and metastasis of HCC by inhibiting DUB3-mediated deubiquitylation of Snail protein [46]. In the current study, we elucidate that CDK4/6-mediated phosphorylation of DUB3 promotes HCC progression through stabilizing YAP1, supported by the following evidence. Firstly, we show that DUB3 directly interacts with, deubiquitinates and stabilizes YAP1 in an enzymatic activity-dependent manner (Figs. 3D–F and 4). Secondly, the genetic ablation of DUB3 markedly reduces the protein levels of YAP1 and its downstream targets, inhibits cell proliferation and tumor growth, and enhances chemosensitivity in HCC (Figs. 5 and S4). Furthermore, reconstitution of YAP1 expression significantly reverses the phenotypes observed with DUB3 depletion (Fig. 5), suggesting that DUB3 promotes HCC progression in a YAP1-dependent manner. Lastly, we confirm that CDK4/6 binds and phosphorylates DUB3 at Ser41, while both the CDK4/6 inhibitor Abemaciclib and the DUB3 S41A mutant nearly abolish DUB3 phosphorylation at Ser41 (Fig. 6A–D). Importantly, we confirm that CDK4/6-mediated phosphorylation of DUB3 is critical for YAP1 stabilization, thereby promoting YAP1-driven cell proliferation and chemo-resistance in HCC. Inhibiting this phosphorylation event by pharmacological inhibition of CDK4/6 or reconstitution of the DUB3 S41A mutant fails to promote the malignant phenotype described above (Fig. 6E–L). Given that YAP1 and TAZ are homologues with overlapping functions as downstream effectors of the Hippo pathway [4], we also observed that co-knockdown of CDK4/6, treatment with Abemaciclib or DUB3 depletion could reduce TAZ protein levels, likely due to increased TAZ polyubiquitination (Figs. S3 and S4). However, since our study primarily focused on YAP1, the influence of the CDK4/6-DUB3 axis on TAZ remains unclear and requires further exploration in future studies.

In conclusion, our study provides significant preclinical evidence that targeting the CDK4/6-DUB3 axis is an effective therapeutic strategy to destabilize YAP1 and suppress HCC progression. Further research will focus on investigating the correlation and prognostic implications of the CDK4/6-DUB3-YAP1 axis in human HCC samples. Importantly, the oncogenic roles of CDK4/6 and YAP1 have been identified not only in HCC, but also in many other cancers, including breast cancer [22, 47], esophageal cancer [34, 48], pancreatic ductal adenocarcinoma [35, 49]. The CDK4/6-DUB3-YAP1 axis may represent a common regulatory mechanism with broader implications for the treatment of multiple cancers, warranting further investigation.

## Materials and methods

### Cell Culture, plasmids and antibodies

Cancer cell lines HCCLM3, HepG2 and human embryonic kidney (HEK) 293 T cell line were purchased from American Type Culture Collection. All cell lines were mycoplasma-free and authenticated by short tandem repeat DNA profiling analysis. HepG2, Huh-7, Hep3B, PLC/PRF/5 and HEK293T cells were cultured in DMEM (Gibco) medium supplemented with 10% FBS (ExCell Bio). HCCLM3 cells were cultured in DMEM (Gibco) medium supplemented with 15% FBS (ExCell Bio).

*YAP1* and *DUB3* were cloned into pIRES (containing FLAG and S tag), pLV.3-FLAG, pLV.6-GFP, pGEX4T-1 and pET30a vectors. All site mutants were generated by site-directed mutagenesis (TOYOBO) and verified by sequencing. Targeting sequences for YAP1 shRNAs are 5′-CAGGTGATACTATCAACCAAA-3′ and 5′-GCCACCAAGCTAGATAAAGAA-3′; Sequences for CDK4 and CDK6 shRNAs are 5′-GAGATTACTTTGCTGCCTTAA-3′ and 5′-CAGATGTTGATCAACTAGGAA-3′; Sequences for DUB3 shRNAs are 5′- GGGAAATACCTGCTACGAGAA -3′ and 5′- GCACCTTAGACCACTGGAAAT′.

Antibodies against YAP1 (66900-1-Ig, dilution: 1:1,000), TAZ (23306-1-AP, dilution: 1:1,000), CYR61 (26689-1-AP, dilution: 1:1,000), CTGF (25474-1-AP, dilution: 1:1,000) and DUB3 (26143-1-AP, dilution: 1:300) were purchased from Proteintech Group Co. Anti-FLAG (F1804, dilution: 1:1,000), anti-HA (H3663, dilution: 1:1,000) and anti-*β*-actin (A1978, dilution: 1:5,000) antibodies were purchased from Sigma-Aldrich Co. Anti-ubiquitin (sc-8017, dilution: 1:300) antibody was purchased from Sigma-Aldrich. Anti-CDK substrate (9477S, dilution: 1:500), Anti-CDK4 (12790, dilution: 1:1,000) and Anti-CDK6 (13331, dilution: 1:1,000) antibodies were purchased from Cell Signaling Technology. Light chain or heavy chain specific IPKine™ HRP were purchased from Abbkine Scientific Co.Ltd. Anti-HA affinity gel and anti-Flag affinity gel were purchased from Beyotime Biotechnology and Sigma-Aldrich Co., respectively.

### Small molecular compounds screening by luciferase reporter assay

The in-house library of small molecule compounds contains 374 compounds supplied in 96-well plates. HCCLM3 cells (3 × 10^6^) were co-transfected with 3 μg of 8 × GTIIC-luciferase plasmid [50], a YAP1/TAZ-responsive luciferase reporter, and 300 ng of Renilla luciferase plasmid as an internal control. After 24 hours, the cells were seeded in a 96-well plate at a density of 4,000 cells/well. Subsequently, the cells were treated with either vehicle or each compound at a final concentration of 10 μM for 24 hours (*n* = 3). The firefly and Renilla luciferase values were measured using Dual-Lumi^TM^ Luciferase Reporter Gene Assay Kit (RG088M, Beyotime Biotechnology) according to the manufacturer’s protocol. Firefly luciferase activity relative to Renilla luciferase activity was calculated for each well. The ratio of firefly to Renilla luciferase activity of each compound was normalized to the DMSO vehicle control.

### Immunoprecipitation

For endogenous interaction, HCCLM3 or HepG2 cells as indicated were lysed in NETN buffer and were incubated for 4 hours with primary antibodies together with protein A/G beads at 4 °C. The immunoprecipitates were subjected to western blotting after washing beads for four times. For ubiquitination assay, cells were transfected with the indicated plasmids and treated with 10 mM MG-132 for 10 hours. Cells were dissolved in 100 μl of 62.5 mM Tris-HCl (pH 6.8), 10% glycerol, 2% SDS, 1 mM iodoacetamide and 20 mM NEM, boiled for 15 minutes, diluted 10 times with NETN buffer containing protease inhibitors, 1 mM iodoacetamide and 20 mM NEM, and centrifuged to remove cell debris. Cell lysates were incubated with HA-protein agaroses or anti-Flag affinity gel at 4 °C for 2 hours. The immunoprecipitates were washed four times and subjected to western blot.

### Glutathione S-transferase (GST) pull-down assay

GST, GST-DUB3 WT and His-YAP1 proteins were expressed in Escherichia coli strain BL21. GST-fusion proteins were purified using Pierce Glutathione agarose and mixed with purified His-YAP1 at 4 °C for 2 hours. Beads were washed four times and proteins were detected by western blot.

### In vitro ubiquitination assay

Cells were transfected with empty vector or Flag-YAP1 and treated with 10 mM MG-132 for 10 hours. YAP1 in the cell lysate were immunoprecipitated using anti-Flag affinity gel. The recombinant GST-tagged DUB3 WT and C89S mutant proteins were expressed in *Escherichia coli* strain BL21 and purified using Pierce Glutathione Agarose. The proteins were then eluted with GST washing buffer (10 mM GSH and 50 mM Tris-HCl, pH=8.0) and purified with ultrafiltration tube. The ubiquitinated YAP1 protein was then incubated separately with purified GST-DUB3 WT and C89S proteins for 4 hours at 4 °C, followed by western blot analysis.

### Quantitative real-time PCR (qRT-PCR)

RNA extraction from cultured cells was performed using TRIzol reagent (Thermo Scientific, MA, USA), and then RNA was subsequently reverse transcribed to cDNA using a FastKing gDNA Dispelling RT SuperMix (Tiangen, Beijing, China). qRT-PCR analysis was performed using FastFire qPCR PreMix (SYBR Green) with primers against YAP1. All experiments were performed in triplicate with GAPDH as an internal control. All samples were normalized to *GAPDH* mRNA levels. Primer sequences are listed. *GAPDH* Forward: ATGGGGAAGGTGAAGGTCG, Reverse: GGGGTCATTGATGGCAACAATA, *YAP1* Forward: TGTCCCAGATGAACG TCACAGC, Reverse: TGGTGGCTGTTTCACTGGAGCA.

### Cell proliferation assay

HCCLM3 (2 × 10^4^) or HepG2 (2 × 10^4^) cells were seeded in 6-well plates and trypsinized with 0.25% trypsin at 37 °C. Cell pellets were collected by centrifugation, washed by PBS, re-suspended in PBS and counted under microscope.

### Animal studies

For subcutaneous xenografting, HCCLM3 cells (5 × 10^6^ cells/mouse) were injected subcutaneously in the flank of six-week-old female BALB/c nude mice (*n* = 6). When tumors reached around 150 mm^3^ in size, mice were administered with saline, Abemaciclib (75 mg/kg, oral gavage 4 days a week for 4 weeks), cisplatin (5 mg/kg intraperitoneal twice per week), or the combination of Abemaciclib and cisplatin. After mice were sacrificed, tumors were surgically removed and weighed. All animal experiments were performed in accordance with a protocol approved by the Institutional Animal Care and Use Committee (IACUC) in Jinan University (20240220-21).

### Statistical analysis

Cell proliferation and survival experiments were independently performed for three times, following the principle of repeatability. In the animal study, data are represented as the mean ± S.D. of six mice. Statistical analyses were performed using GraphPad Prism software version 9.3. One-way ANOVA analysis and Tukey’s test or *t-test* were used to compare results.

## Supplementary information

Supplementary Figures and Figure Legends

Figure S1

Figure S2

Figure S3

Figure S4

Original western blots

## Data Availability

All data generated or analyzed during this study are available from the corresponding authors upon reasonable requests.
