# Supplementary material for: CDK4/6-mediated phosphorylation of DUB3 promotes YAP1 stability and hepatocellular carcinoma progression
Source: Cell Death Discov. 2025 Apr 30;11:212. doi: 10.1038/s41420-025-02493-x (PMC12044017; doi:10.1038/s41420-025-02493-x)
Supplement: Supplementary file 1 — Supplementary Figures and Figure Legends [file 41420_2025_2493_MOESM1_ESM.docx]

**Supplementary Information**

**CDK4/6-mediated phosphorylation of DUB3 promotes YAP1 stability and hepatocellular carcinoma progression**

**Content: Supplementary Figures and Figure Legends**

**
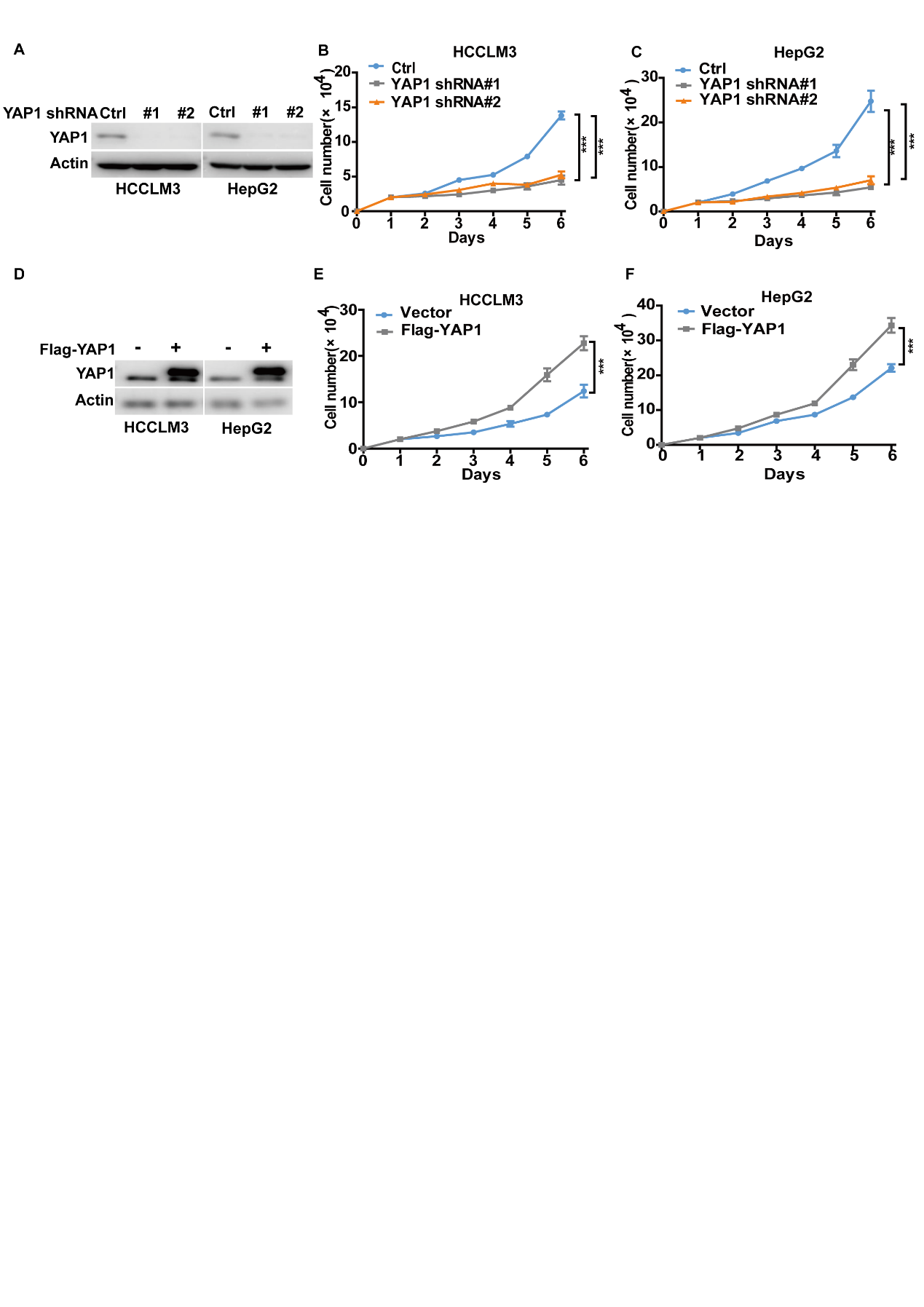
**

**Figure S1. YAP1 plays an oncogenic role in hepatocellular carcinoma. A** HCCLM3 and HepG2 cells stably expressing control (Ctrl) or YAP1 shRNAs (#1 and #2) were generated and western blot was performed with indicated antibodies. **B, C** Cell proliferation assay was performed from cells in (**A**). Results represent the mean ± S.D. of three independent experiments. ****p* < 0.001, Ctrl vs YAP1 shRNA #1, Ctrl vs YAP1 shRNA #2. **D** HCCLM3 and HepG2 cells stably expressing vector or Flag-YAP1 were generated. Western blot was performed with indicated antibodies. **E, F** Cell proliferation assay was performed from cells in (**D**). Results represent the mean ± S.D. of three independent experiments. ****p* < 0.001, vector vs Flag-YAP1.


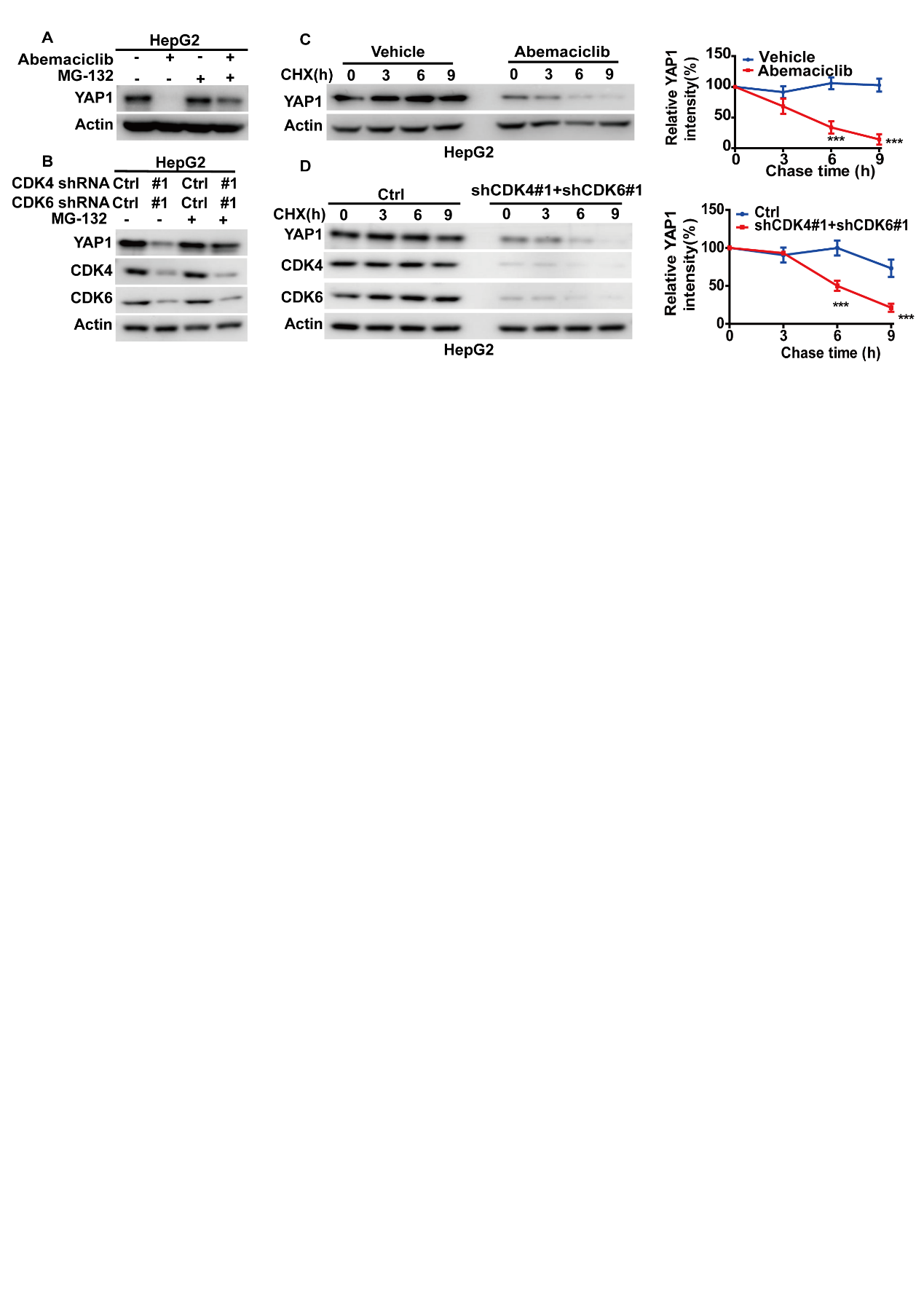


**Figure S2. CDK4/6 regulates YAP1 protein stability.** **A** HepG2 Cells were pretreated with vehicle or Abemaciclib and treated with either vehicle or MG-132 (10 μM) for an additional 24 hours. Western blot was performed with indicated antibodies. **B** HepG2 cells stably expressing control (Ctrl) or CDK4 shRNA#1 + CDK6 shRNA#1 were treated with vehicle or MG-132 and western blot was performed with indicated antibodies. **C** Cycloheximide pulse-chase assay was performed in HepG2 cells treated with vehicle or Abemaciclib; the relative level of YAP1 to *β*-actin was measured by image J. The results represent mean ± S.D. from three independent experiments; ****p* <0.001. **D** Cycloheximide pulse-chase assay was performed in HepG2 cells expressing control or CDK4 shRNA#1 + CDK6 shRNA#1; the relative level of YAP1 to *β*-actin was measured by image J. The results represent mean ± S.D. from three independent experiments; ****p* <0.001.


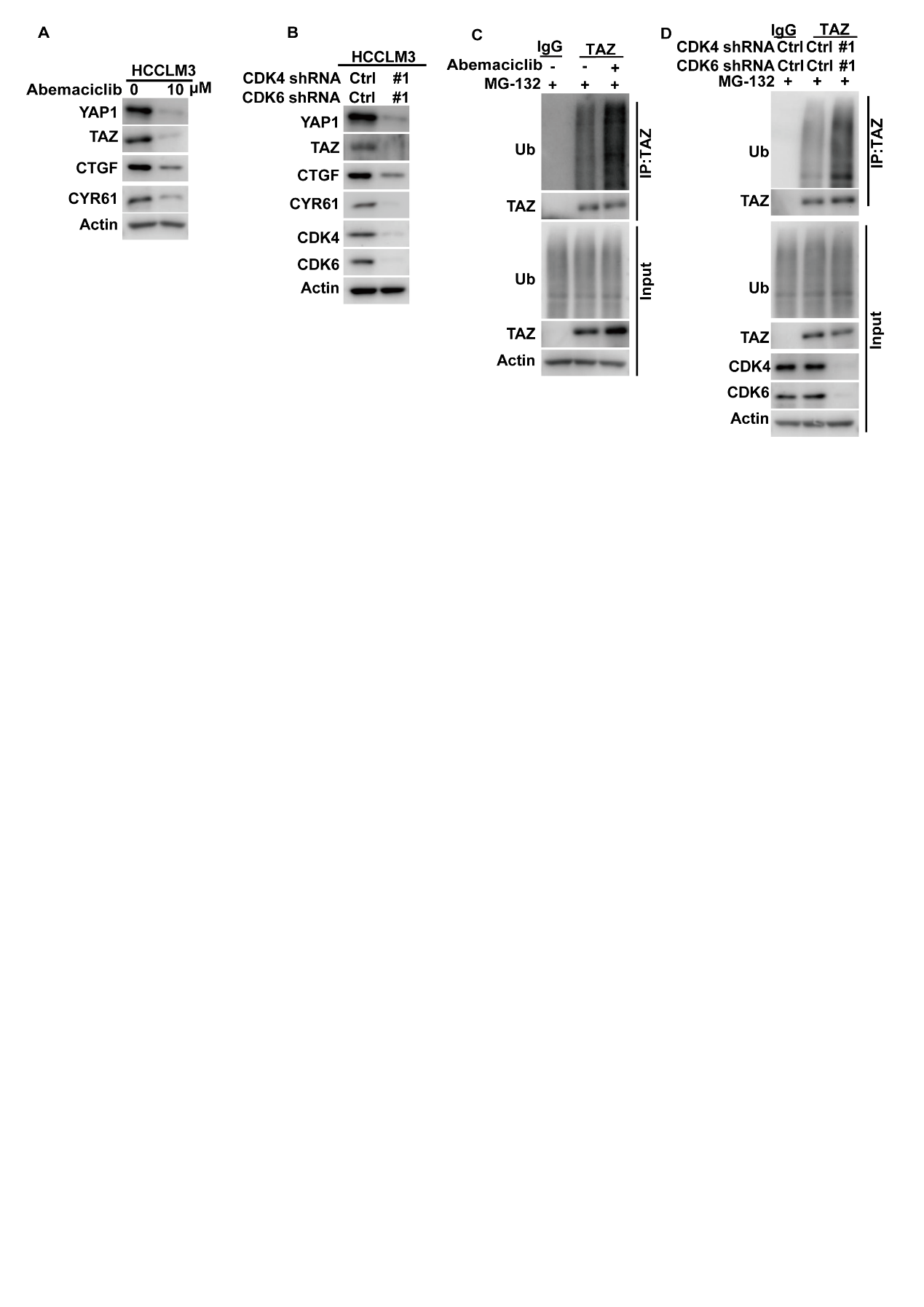


**Figure S3. CDK4/6 regulates TAZ protein levels by affecting its ubiquitination.** **A** HCCLM3 cells were treated with Abemaciclib at indicated concentrations for 24 hours and western blot was performed with indicated antibodies. **B** HCCLM3 cells stably expressing control (Ctrl) or CDK4 shRNA#1 + CDK6 shRNA#1 were generated and western blot was performed with indicated antibodies. **C** Cells treated with vehicle or Abemaciclib for 24 hours in the presence of MG-132 (10 μM). Cell lysates were immunoprecipitated with IgG or anti-TAZ, and the polyubiquitylated TAZ was detected by anti-ubiquitin antibody. **D** Cells stably expressing control or CDK4/6 shRNAs treated with MG-132 (10 μM) for 10 hours. Cell lysates were subjected to immunoprecipitation with IgG or anti-TAZ, and the polyubiquitylated TAZ was detected by anti-ubiquitin antibody.


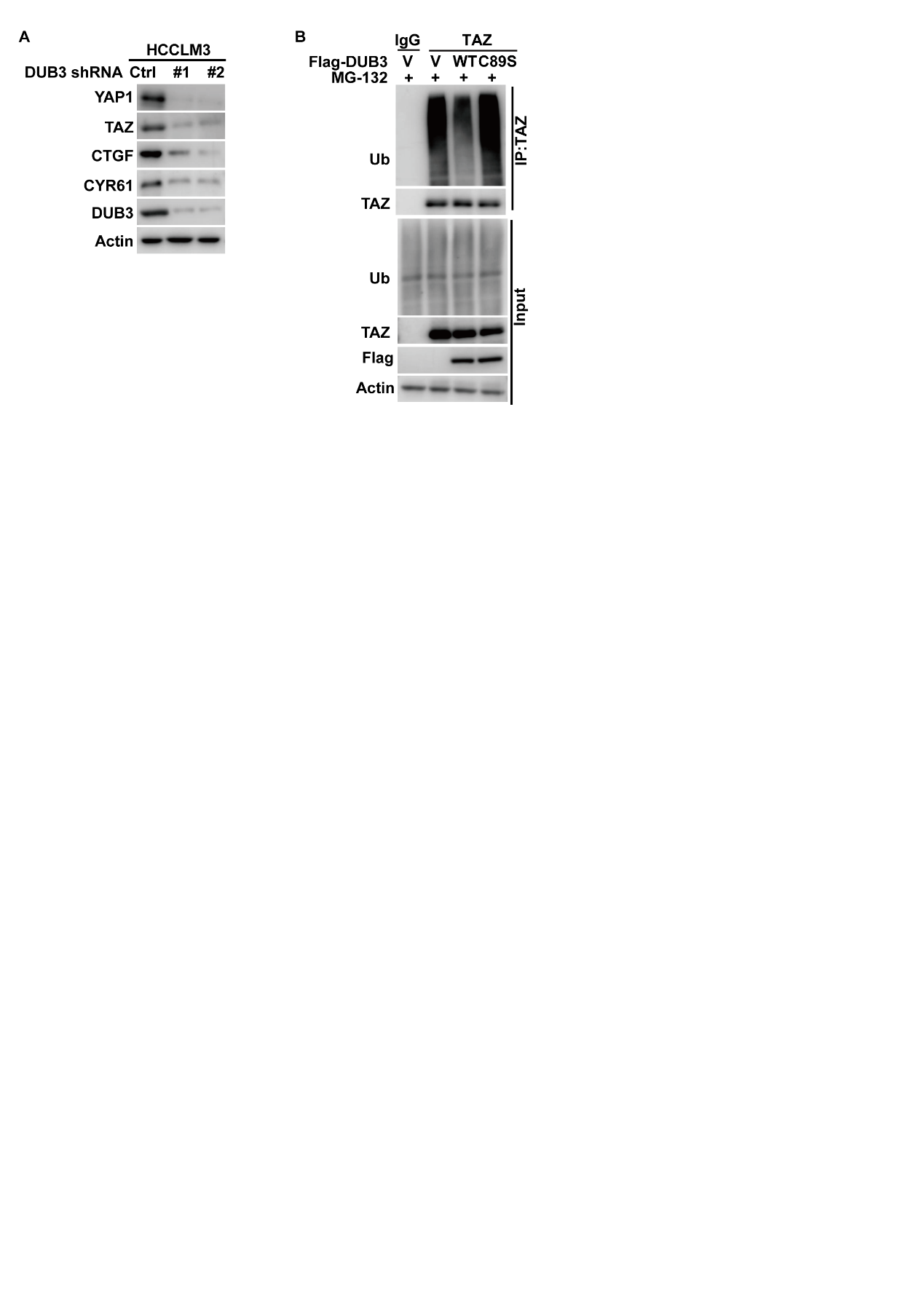


**Figure S4.** **DUB3 regulates TAZ protein levels by affecting its ubiquitination.** **A** HCCLM3 cells stably expressing control (Ctrl) or DUB3 shRNAs (#1 and #2) were generated and western blot was performed with indicated antibodies. **B** Cells were transfected with vector (V) or Flag-DUB3 WT or Flag-DUB3 C89S and then treated with MG-132 (10 μM) for 10 hours. Cell lysates were subjected to immunoprecipitation with IgG or anti-TAZ, and the polyubiquitylated TAZ was detected by anti-ubiquitin antibody.
