# Supplementary figures and images for: CDK4/6-mediated phosphorylation of DUB3 promotes YAP1 stability and hepatocellular carcinoma progression
Source: Cell Death Discov. 2025 Apr 30;11:212. doi: 10.1038/s41420-025-02493-x (PMC12044017; doi:10.1038/s41420-025-02493-x)

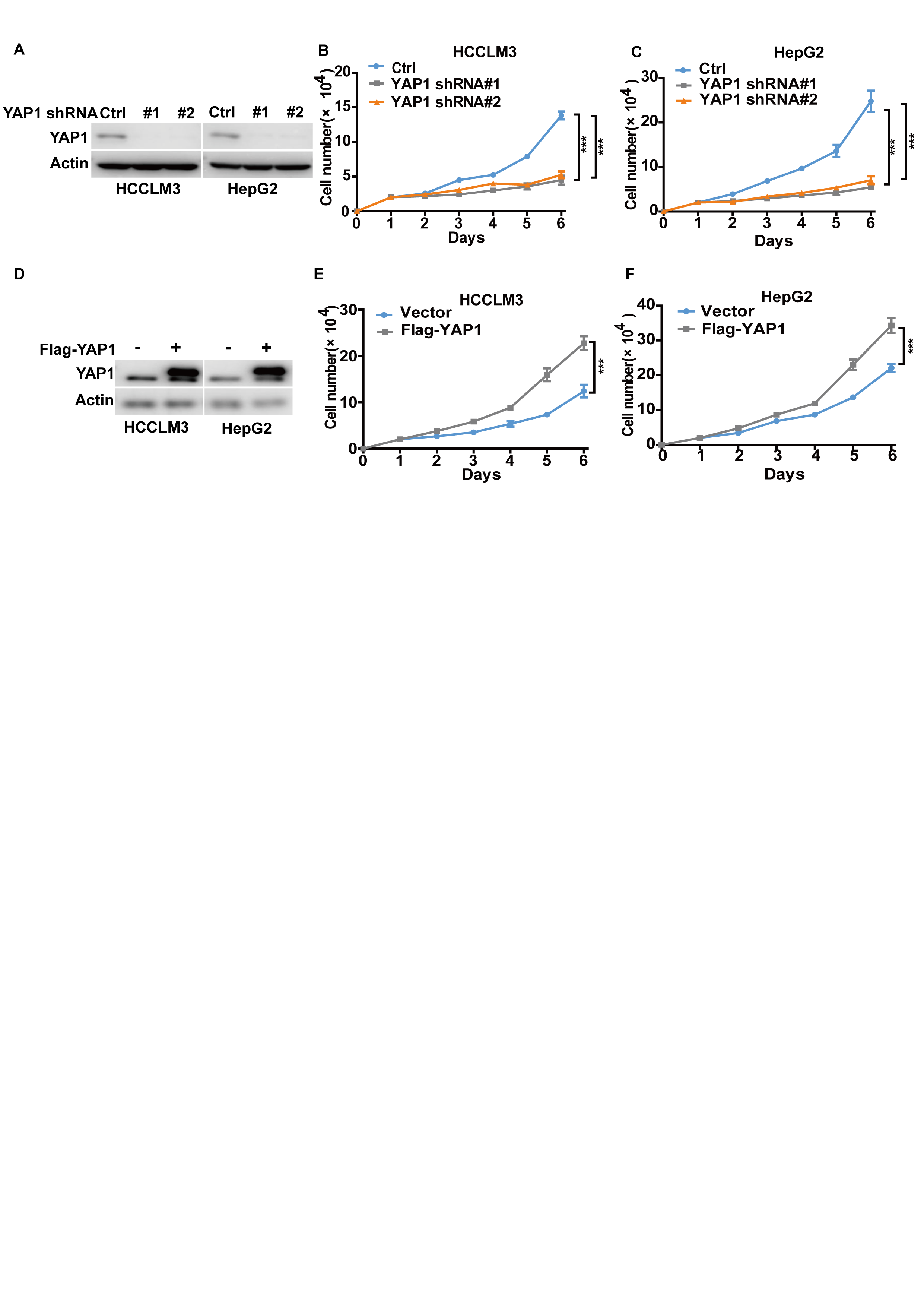

Supplement: Supplementary file 2 — Figure S1 [file 41420_2025_2493_MOESM2_ESM.tif]

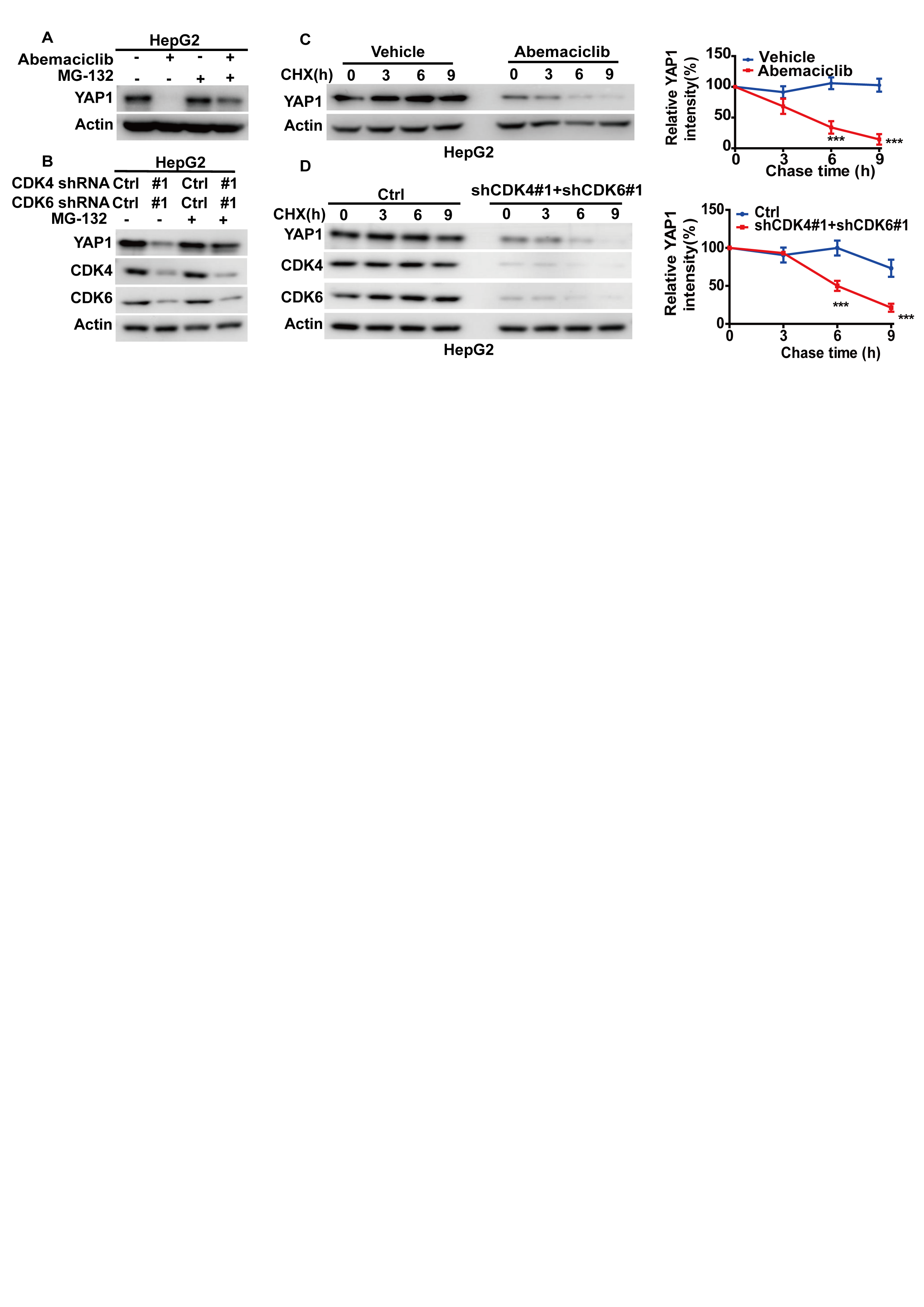

Supplement: Supplementary file 3 — Figure S2 [file 41420_2025_2493_MOESM3_ESM.tif]

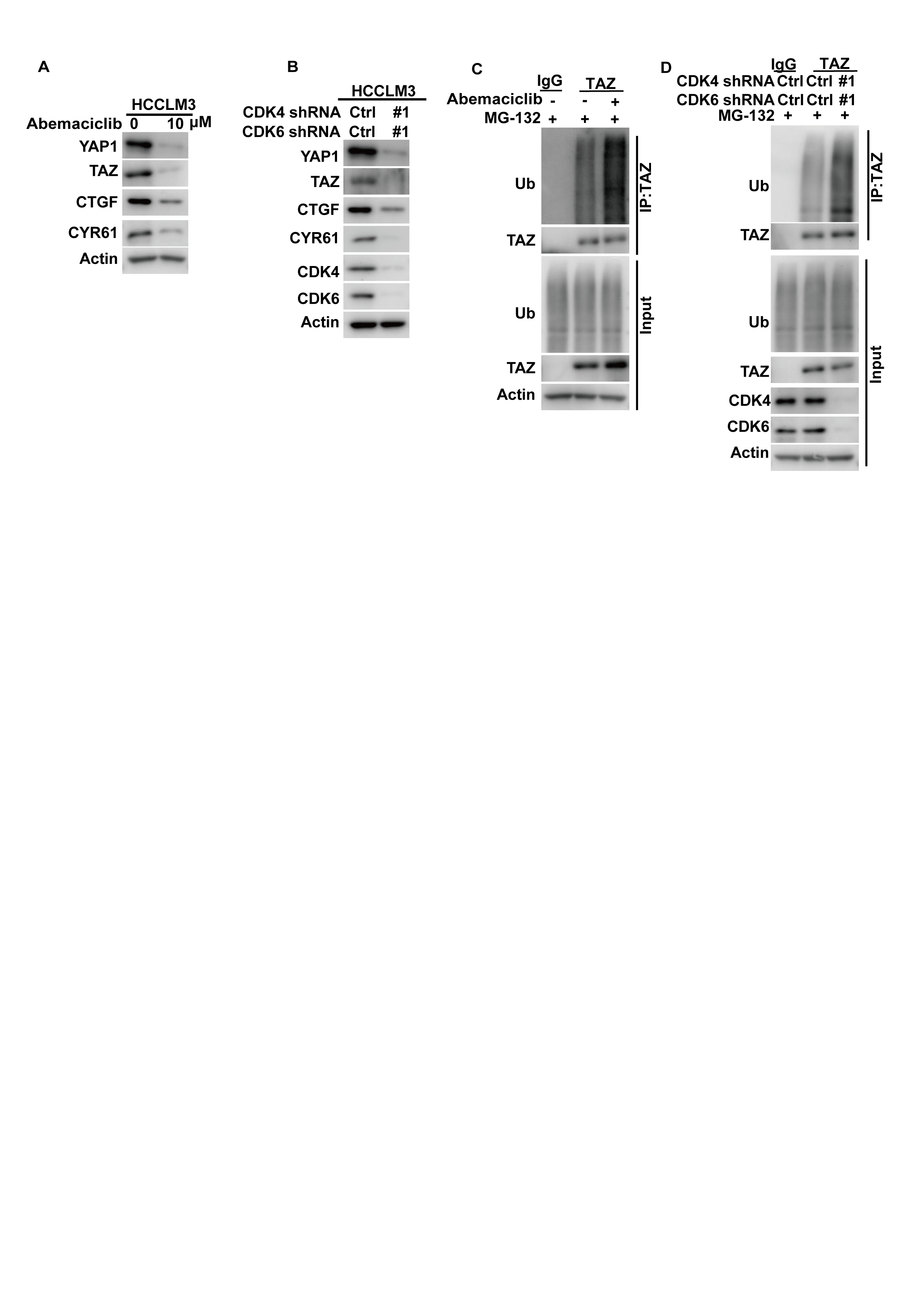

Supplement: Supplementary file 4 — Figure S3 [file 41420_2025_2493_MOESM4_ESM.tif]

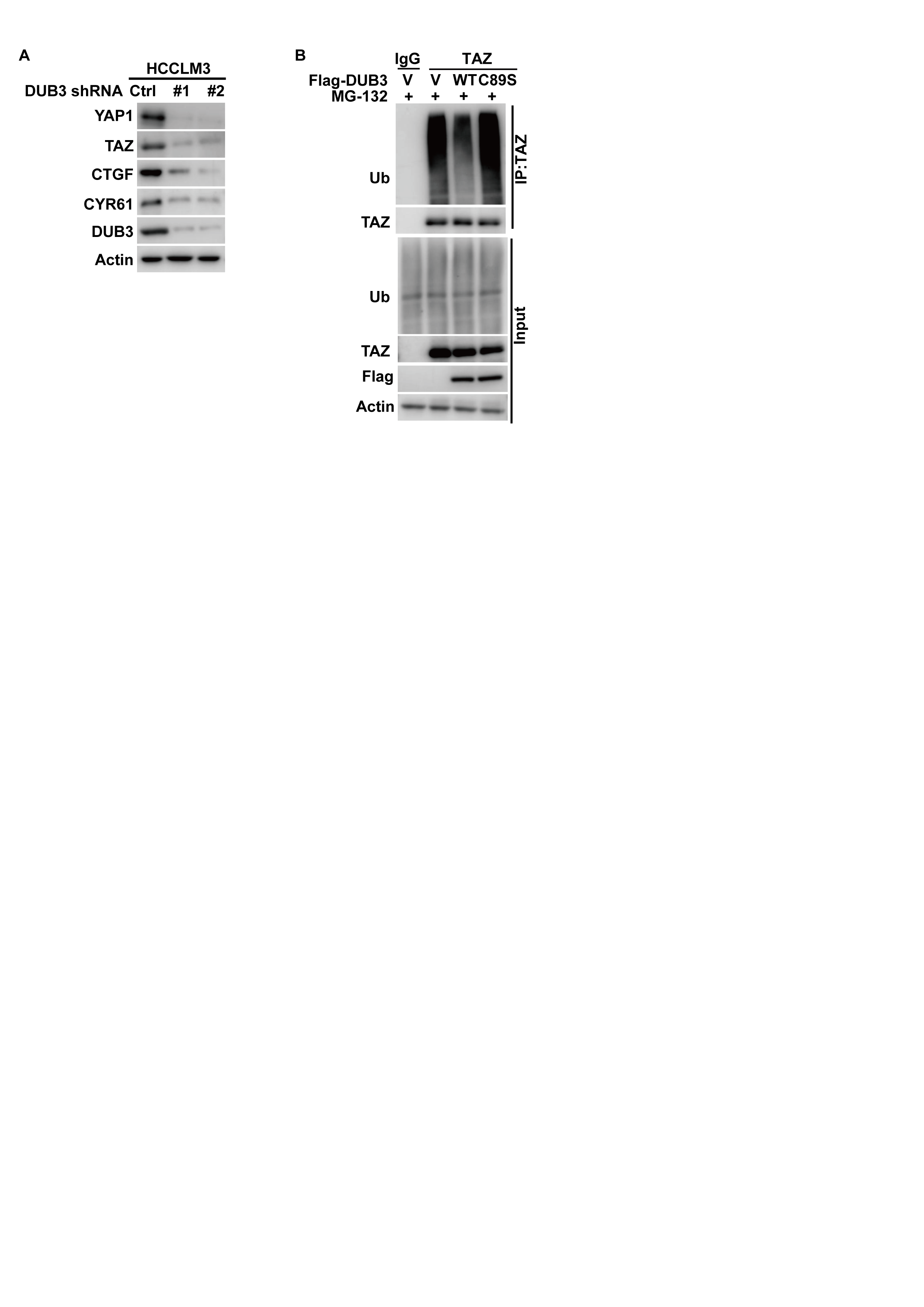

Supplement: Supplementary file 5 — Figure S4 [file 41420_2025_2493_MOESM5_ESM.tif]
