# Supplementary material for: CDK4/6-mediated phosphorylation of DUB3 promotes YAP1 stability and hepatocellular carcinoma progression
Source: Cell Death Discov. 2025 Apr 30;11:212. doi: 10.1038/s41420-025-02493-x (PMC12044017; doi:10.1038/s41420-025-02493-x)
Supplement: Supplementary file 6 — Original western blots [file 41420_2025_2493_MOESM6_ESM.pdf]

CDK4/6-mediated phosphorylation of DUB3 promotes YAP1 stability and hepatocellular carcinoma progression

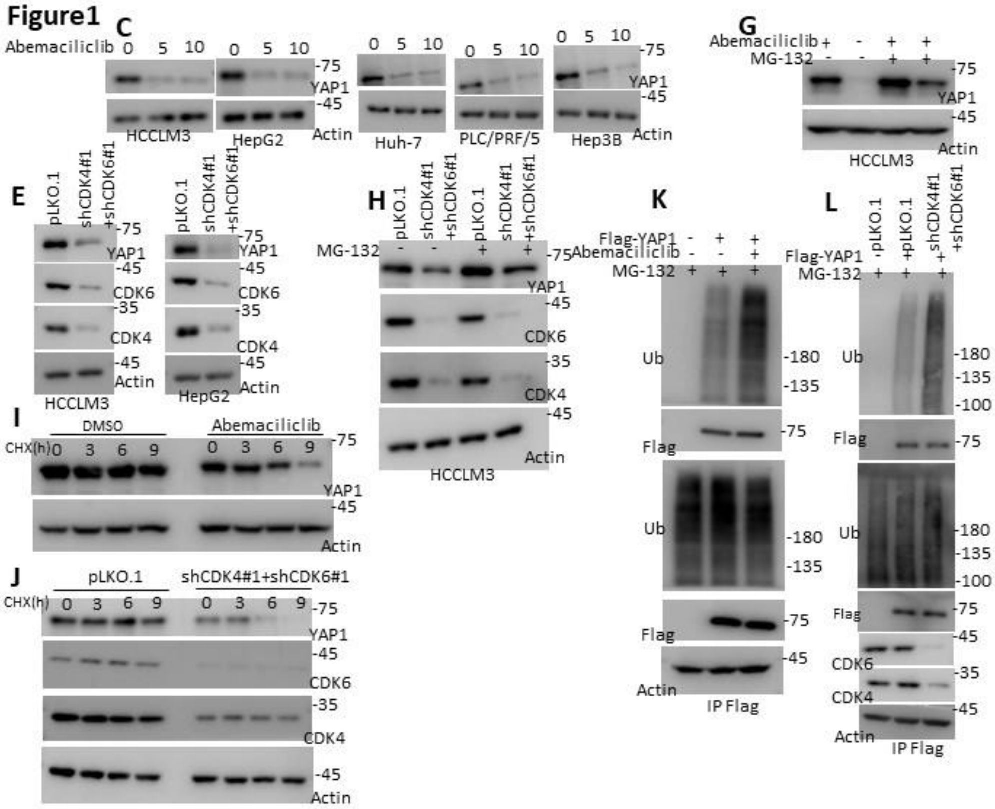

Original scan of the blots presented in the main text. Related to Figure1.

Figure2

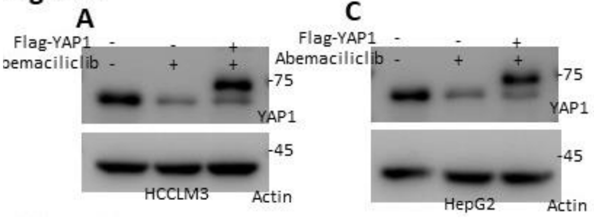

Original scan of the blots presented in the main text. Related to Figure2.

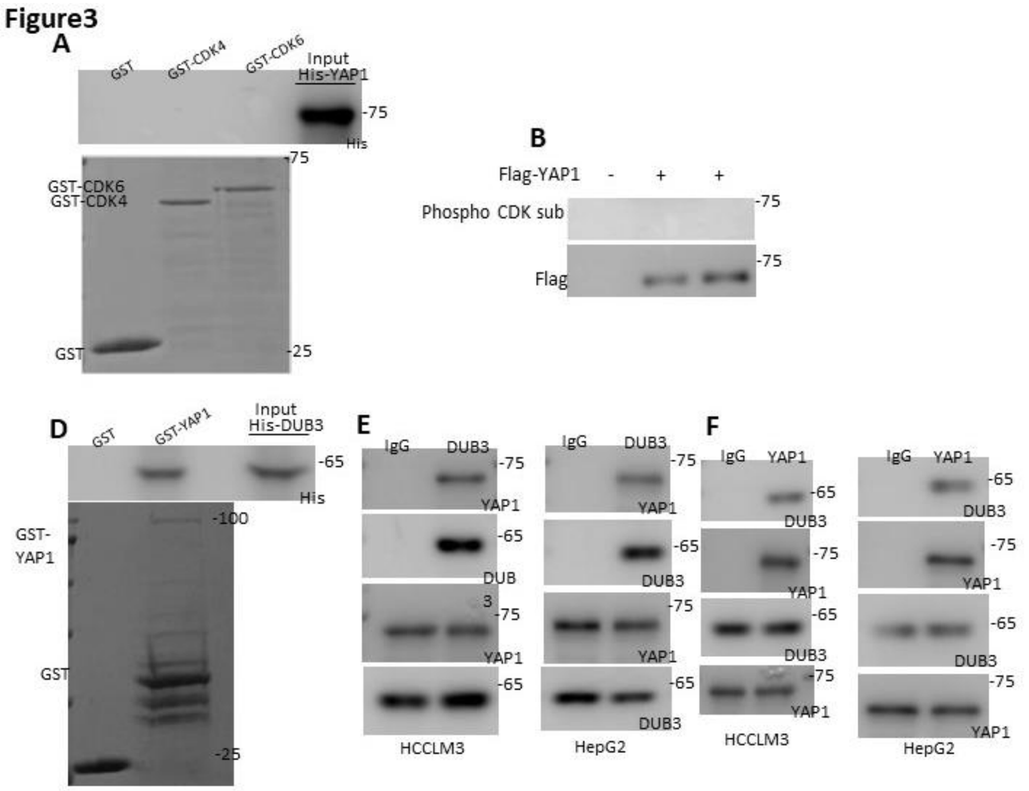

Original scan of the blots presented in the main text. Related to Figure 3.

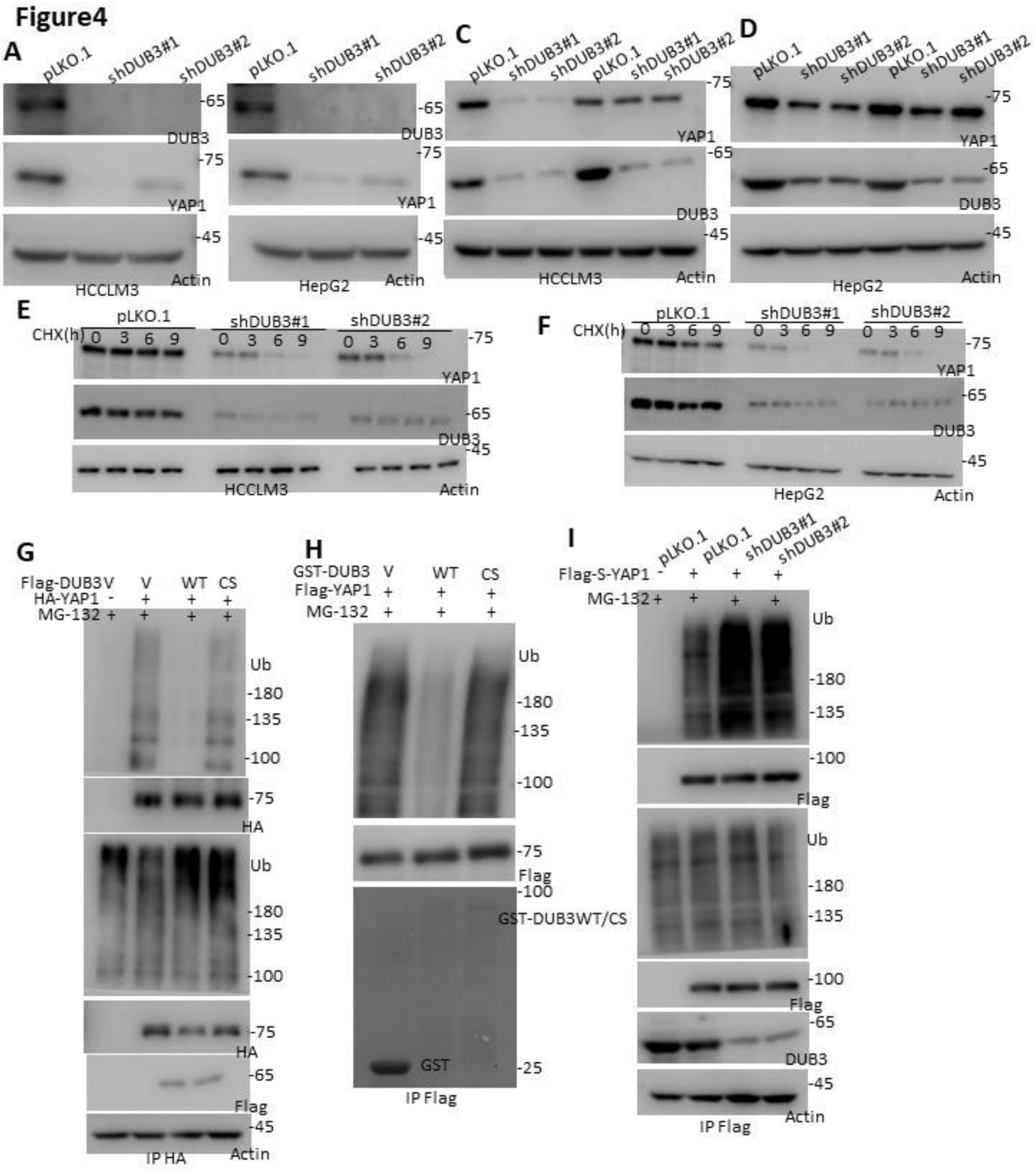

Original scan of the blots presented in the main text. Related to Figure 4.

Figure5

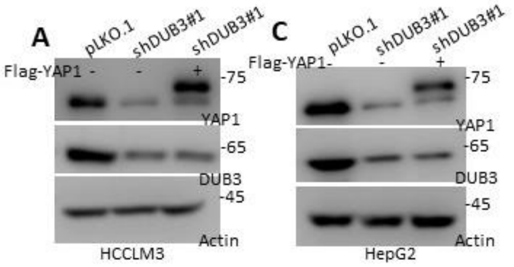

Original scan of the blots presented in the main text. Related to Figure 5.

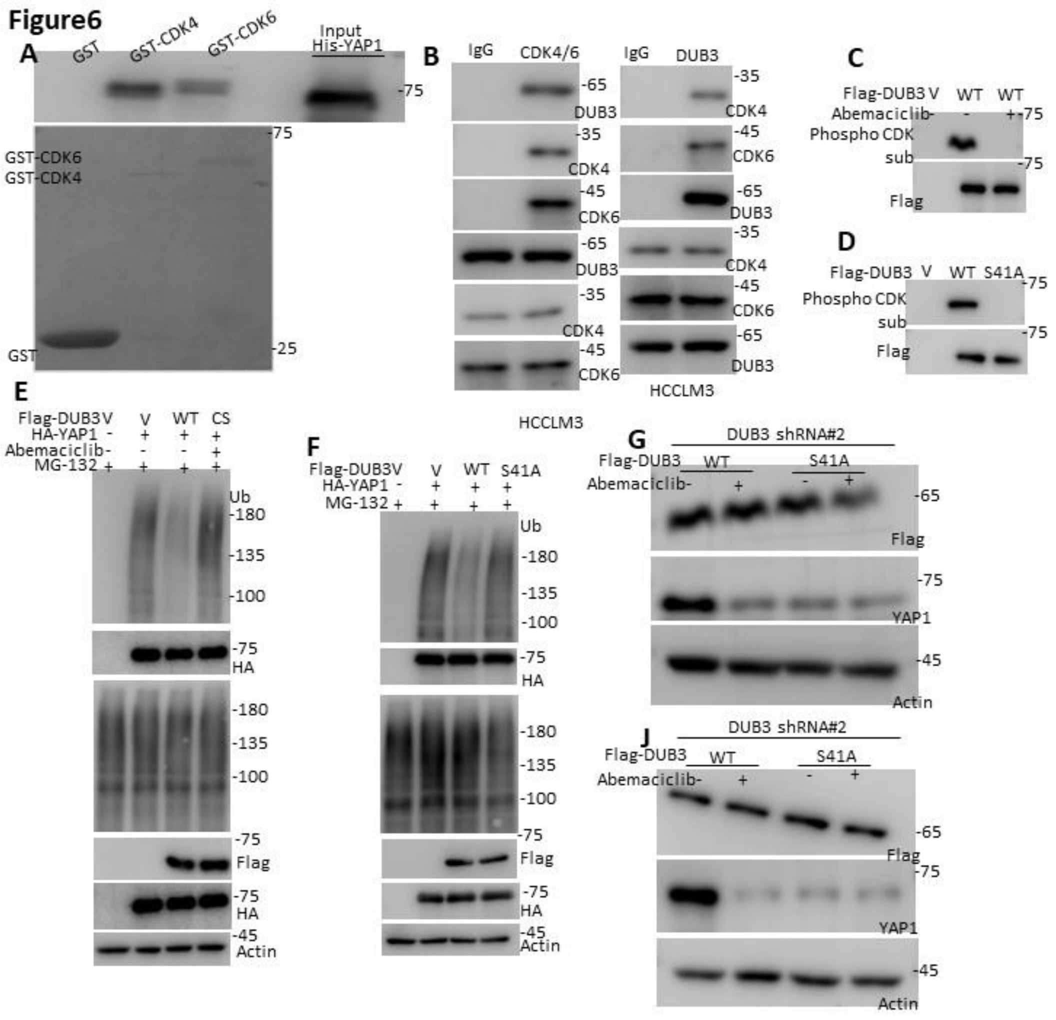

Original scan of the blots presented in the main text. Related to Figure 6.

FigureS1

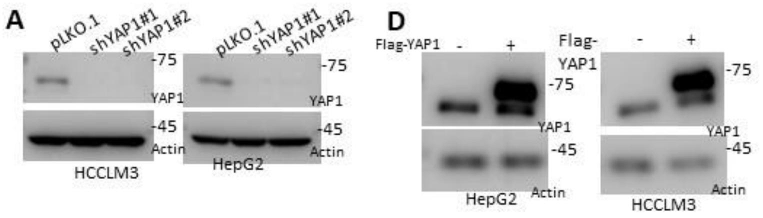

FigureS2

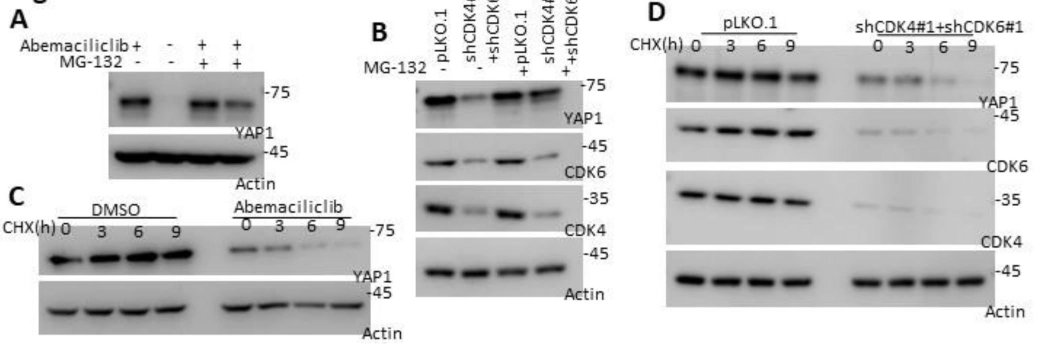

Original scan of the blots presented in the main text. Related to Figure S1 and Figure S2.

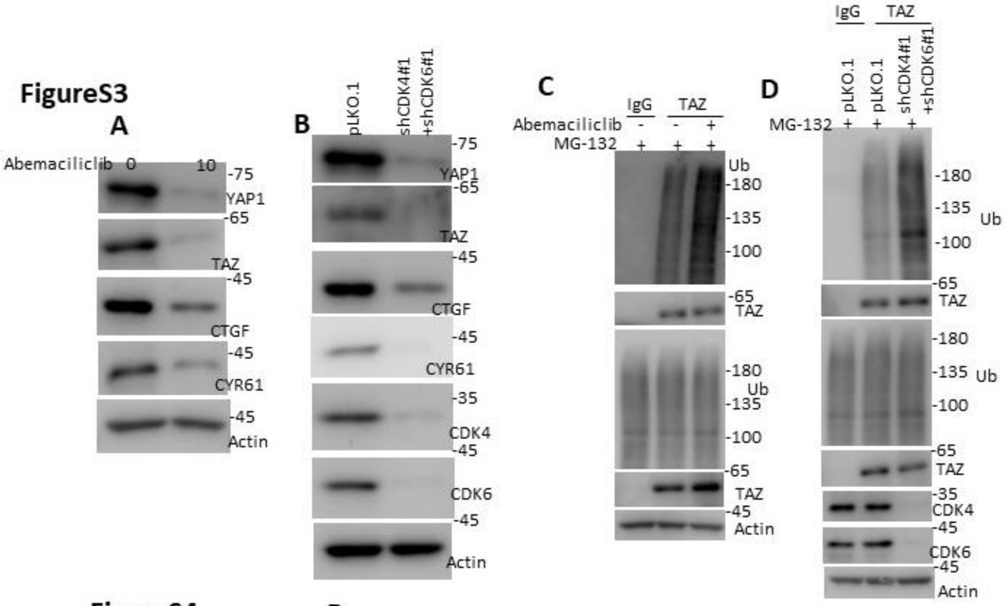

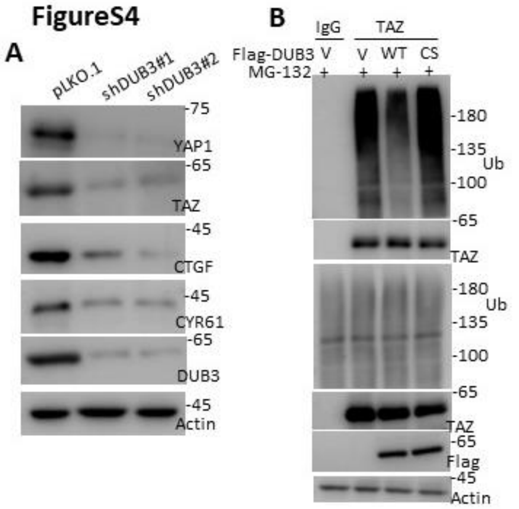

Original scan of the blots presented in the main text. Related to Figure S3 and Figure S4.
